# Intracellular delivery of anti-BCR/ABL antibody by PLGA nanoparticles suppresses the oncogenesis of chronic myeloid leukemia cells

**DOI:** 10.1186/s13045-021-01150-x

**Published:** 2021-09-06

**Authors:** Guoyun Jiang, Zhenglan Huang, Ying Yuan, Kun Tao, Wenli Feng

**Affiliations:** 1 Department of Clinical Hematology, School of Laboratory Medicine, Chongqing Medical University, No. 1, Yixueyuan Road, Yuzhong District, Chongqing, 400016 China; 2 Department of Respiratory and Critical Care Medicine, The First Affiliated Hospital of Chongqing Medical University, No. 1, Youyi Road, Yuzhong District, Chongqing, 400016 China; 3 Department of Immunology, College of Basic Medical Science, Chongqing Medical University, Chongqing, China

**Keywords:** Chronic myeloid leukemia, Targeted therapy, Intracellular delivery of antibodies, PLGA nanoparticles, BCR/ABL oncoprotein

## Abstract

**Background:**

The pathogenesis of chronic myeloid leukemia (CML) is the formation of the BCR/ABL protein, which is encoded by the bcr/abl fusion gene, possessing abnormal tyrosine kinase activity. Despite the wide application of tyrosine kinase inhibitors (TKIs) in CML treatment, TKIs drug resistance or intolerance limits their further usage in a subset of patients. Furthermore, TKIs inhibit the tyrosine kinase activity of the BCR/ABL oncoprotein while failing to eliminate the pathologenic oncoprotein. To develop alternative strategies for CML treatment using therapeutic antibodies, and to address the issue that antibodies cannot pass through cell membranes, we have established a novel intracellular delivery of anti-BCR/ABL antibodies, which serves as a prerequisite for CML therapy.

**Methods:**

Anti-BCR/ABL antibodies were encapsulated in poly(d, l-lactide-*co*-glycolide) nanoparticles (PLGA NPs) by a double emulsion method, and transferrin was labeled on the surface of the nanoparticles (Ab@Tf-Cou6-PLGA NPs). The characteristics of nanoparticles were measured by dynamic light scattering (DLS) and transmission electron microscopy (TEM). Cellular uptake of nanoparticles was measured by flow cytometry (FCM). The effect of nanoparticles on the apoptosis and proliferation of CML cells was testified by FCM and CCK-8 assay. In addition, the anti-cancer impact of nanoparticles was evaluated in mouse models of CML.

**Results:**

The results demonstrated that the Ab@Tf-Cou6-PLGA NPs functioned as an intracellular deliverer of antibodies, and exhibited an excellent effect on degrading BCR/ABL oncoprotein in CML cells via the Trim-Away pathway. Treatment with Ab@Tf-Cou6-PLGA NPs inhibited the proliferation and induced the apoptosis of CML cells in vitro as well as impaired the oncogenesis ability of CML cells in vivo.

**Conclusions:**

In conclusion, our study indicated that this approach achieved safe and efficient intracellular delivery of antibodies and degraded BCR/ABL oncoprotein via the Trim-Away pathway, which provides a promising therapeutic strategy for CML patients, particularly those with TKI resistance.

**Supplementary Information:**

The online version contains supplementary material available at 10.1186/s13045-021-01150-x.

## Background

Chronic myeloid leukemia (CML) is a myeloproliferative malignancy arising from the hematopoietic stem cell (HSC) compartment. The pathogenic mechanism is the formation of the bcr/abl oncogene, caused by the translocation of chromosome 9 and chromosome 22 [1]. The BCR/ABL oncoprotein, which is encoded by the bcr/abl oncogene, maintains constitutive tyrosine kinase activity, induces the activation of downstream signaling pathways, promotes the proliferation and inhibits the apoptosis of CML cells [2–4]. Tyrosine kinase inhibitors (TKIs) show remarkable success with high response rates, especially the first-generation TKI imatinib, which is considered to be the first-line for CML therapy. TKIs intolerance or resistance affects approximately 25% of CML patients [5, 6]. Mutations are the most common cause of TKIs resistance [7, 8]. For patients who fail to adequately respond to TKIs treatment, allogeneic bone marrow transplantation (HSCT) offers the possibility of long-term survival [9, 10]. However, patients who receive HSCT treatment tend to have a risk of graft-versus-host disease [11]. Hence, new choices for CML treatment have emerged, such as using the CRISPER techniques to knock out the fusion gene [12], the adoptive immunotherapy [13], and the proteolysis targeting chimera (PROTAC) technology [14], as well as more methods on the way [15].

In the past decades, therapeutic antibodies have been widely used in cancer therapy. Since the first antibody-drug was approved by FDA in 1986, 91 antibodies have been approved by the FDA by the end of 2019 [16]. Generally, therapeutic antibodies are mostly used to target specific proteins on the surface of cells [17], while the key pathogenic BCR/ABL protein of CML is located inside the cells, which limits the use of antibodies for CML target treatment. The Trim-Away pathway has recently been reported as a rapid and acute endogenous protein degradation method that degrades endogenous protein with specific intracellular antibodies via the TRIM21 and the proteasome [18]. Tripartite motif containing 21 (TRIM21) is a multiple domain Fc receptor with a ubiquitin ligase function which is expressed in most histogenesis cells [19, 20]. TRIM21 belongs to the large family of tripartite motif proteins, it can play an antiviral role through antibody-dependent intracellular neutralization (ADIN) and participate in the innate immune pathway [21]. There are four domains in TRIM21. Among them, the PRYSPRY domain of the C-terminal can specifically combine with the Fc segment of antibodies, and the RING domain of the N-terminal has E3 ubiquitin ligase activity, which can transport the compound to the proteasome for degradation [22, 23]. In brief, the intracellular antibodies can specifically combine with endogenous protein and the complex can be efficiently degraded via the Trim-Away pathway [18]. Therefore, transportation of anti-BCR/ABL antibodies into CML cells can efficiently degrade BCR/ABL proteins via the Trim-Away pathway, thus blocking the downstream signal transduction of BCR/ABL tyrosine kinases, eventually eliminating the pathogenesis of CML cells.

Since the antibodies themselves cannot penetrate the cell membrane, which limits their intracellular applications. To overcome this problem, numerous antibody intracellular delivery methods have been proposed, including electroporation, magnetic transfer, cell microinjection, cell-penetrating peptides, viral vectors, and nanoparticles (NPs) [24, 25]. Notably, the nanoparticle has been identified as the most promising therapeutic application method due to its high maneuverability, extremely low cytotoxicity, remarkable biocompatibility, strong drug loading ability, superior transportation efficiency, and extremely high stability [26, 27]. PLGA has been widely used to formulate nanoparticles due to its biodegradability and biocompatibility. And it has already been approved by the FDA [28, 29]. Therefore, anti-BCR/ABL antibody-loaded PLGA NPs were formulated to serve as the intracellular deliverer of antibodies. Coumarin6 (Cou6) was added as the fluorescence labeling for its green fluorescence to be visible [30]. Furthermore, NH_4_HCO_3_ was used to achieve pH-sensitive antibody release, since NH_4_HCO_3_ can decompose into CO_2_, NH_3_, and H_2_O in acidic environments [31–33]. To increase the CML cell targeting, transferrin (Tf) was labeled on the surface of NPs for the high transferrin receptor (TfR) expression level on the CML cell membrane compared with normal cells [34–36]. This strategy can achieve a higher accumulation of antibody availability for CML cells and reduce systemic antibody exposure.

In this study, we encapsulated anti-BCR/ABL antibodies into PLGA NPs and added a transferrin modification to improve the targeting ability of CML cells. We demonstrated that the antibodies can be effectively delivered into CML cells and efficiently degrade the BCR/ABL oncoproteins through the Trim-Away pathway.

## Methods

### Materials

Poly(d, l-lactide-*co*-glycolide) (Resomer® RG 504) and PVA (87–90% hydrolyze) were purchased from Sigma-Aldrich (UK), the anti-BCR/ABL rabbit antibody is an IgG subtype antibody and recognizes the ABL sequence of BCR/ABL oncoprotein (Sangon Biotech, China), Transferrin (≥ 98%) and Coumarin6(≥ 98%) were purchased from Solarbio (China).

### Synthesis of nanoparticles

PLGA NPs were prepared by double emulsion solvent evaporation (w1/o/w2) methods. Shortly, 0.5 mg of anti-BCR/ABL antibodies were mixed with 0.25 mg of NH_4_HCO_3_ in 200 μl of ultrapure water, and the resulting solution was further mixed with 2 ml of chloroform containing 20 mg of PLGA and 10 μg of Cou6. The mixture was homogenized using an ultrasonic probe (40% amplitude) at 4 °C for 2 min. The resulting solution was then mixed with 10 ml of PVA (1%) and sonicated for 6 min under the same conditions. The emulsion was then stirred at room temperature for 4 h to evaporate chloroform. Finally, the antibody-loaded PLGA NPs were collected by centrifugation at 13,300 rpm for 30 min at 4 °C. PVA and un-entrapped antibodies were removed by washing three times, then the NPs were stored at 4 °C.

Transferrin-modified nanoparticles were prepared according to the procedure reported in previous studies [34, 37]. Transferrin was dissolved in Ringer-Hepes buffer at a dose of 10 mg/ml, and an equal dose of nanoparticles was added to the transferrin solution. The mixture was shaken moderately at 4 °C overnight. The unbonded transferrin was eliminated using a 100 KDa ultra-filtration centrifugal tube (Millipore). The transferrin-modified nanoparticles were washed and stored at 4 °C.

### Characterization of nanoparticles

The characterization of nanoparticles was detected using TEM and DLS. The ingredients of Ab@Tf-Cou6-PLGA NPs were further examined by an SDS-PAGE assay. PLGA NPs, transferrin, antibody and Ab@Tf-Cou6-PLGA NPs were denatured with loading buffer and boiled for 5 min. Each sample was added to the 10% SDS–polyacrylamide gel at a dose of 10 μg and ran at 80 V for 1.5 h. Then the gel was stained with Coomassie blue and imaged.

To prove the successful modification of transferrin on the surface of Ab@Tf-Cou6-PLGA NPs, we performed a dot blotting assay. The PLGA NPs, Cou6-PLGA NPs, Tf-Cou6-PLGA NPs, and Ab@Tf-Cou6-PLGA NPs were adjusted to equal concentration, and 2 μl of each sample was dropped on a NC membrane. After drops dried, the membrane was blocked and incubated with anti-Transferrin antibodies (Beyotime biotechnology, China) overnight. The Goat anti-rabbit IgG-HRP was used as a secondary antibody at 1:5000 dilution. Dots were detected by using an ECL western blotting substrate.

### Loading capacity and release studies

To determine the encapsulation efficiency and release rate of antibodies, we synthesized Ab@Cou6-PLGA NPs. Nanoparticles were dissolved in chloroform and shaken for 1 h at room temperature, then the mixture was centrifugated at 13,300 rpm for 30 min at 4 °C. The sediment was harvested and then 1 ml of NaOH solution (0.1 M) was added. The protein precipitation was slowly dissolved in the NaOH solution and finally detected by the BCA protein assay kit. For the releasing rate assay, nanoparticles were dissolved in 1 ml PBS (pH 5.0 or pH 7.4) and kept shaking at 37 °C. The supernatant was collected after centrifugating at 13,300 rpm for 30 min and detected by an enhanced BCA assay kit.

### Blood compatibility assay

To analyze the blood compatibility of nanoparticles, a blood compatibility assay was performed. First, 2 ml of fresh blood from mice was collected and centrifugated at 2000 rpm for 10 min. Then, the supernatant was discarded and the red blood cells (RBCs) were washed with sterile saline solution until the suspension was colorless. Afterward, the RBCs were re-suspended in sterile saline at 2% hematocrit. Then 0.8 ml of RBCs suspension was mixed with 0.2 mL NPs solution of different concentrations, ultrapure water was used as a positive control and saline as a negative control. The mixtures were incubated for 2 h at 37 °C. The mixtures were then centrifugated at 2000 rpm for 10 min, and the suspension was collected and added into a 96-well plate. The absorbance was measured at 540 nm using a microplate reader (Bio-Teck, USA). The percentage of hemolysis was calculated using the following formula:

$${\text{Hemolysis}}\, \left( \% \right) = \frac{{\left( {{\text{Asample}} - {\text{Anegative}}} \right)}}{{\left( {{\text{Apositive}} - {\text{Anegative}}} \right)}} \times 100\%$$

### Protein absorption assay

NPs were diluted with PBS (pH 5.0 and pH7.4) to a final concentration of 0.5 mg/ml. Afterward, dilutions were mixed with bovine serum albumin (BSA, 0.25 mg/ml) and co-incubated in a water bath shaker for 2 h at 37 °C. Then, the supernatant of each sample was collected by centrifuging at 8000 rpm for 10 min and the protein concentration was quantified using the BSA protein assay kit.

### Cell lines

K562 (ATCC), K562/G01, HL-60, NB4 and A569 cells were cultured in RPMI-1640 (Gibco, USA) with 10% fetal bovine serum (Gibco, USA). K562/G01, an imatinib-resistance cell line, was originated in K562 by co-culture with imatinib for months [38]. Cells were cultured in a standard 5% CO_2_ cell incubator at 37 °C.

### Cell uptake mechanism

To verify the cell uptake mechanism, 2 × 10^5^ cells were seeded in a 24-well plate. The cells were incubated at 4 °C for 1 h to inhibit cell membrane activity, or pretreated with or without sucrose (0.45 mM) for 1 h to inhibit cell endocytosis [39]. Afterward, treatment medium was removed and the cells were shifted into a new medium containing Ab@Tf-Cou6-PLGA NPs (200 μg/ml) and incubated for another hour. After washing three times, the cells were harvested for FCM and fluorescence microscope observation.

### Cytotoxicity studies

For cytotoxicity analysis, 3000 cells of each group were seeded in a 96-well plate. Cells were cultured in 0.1 ml RPMI-1640 medium containing 10% fetal bovine serum. Each well was added with 10 μl of CCK-8(BOSTER, China) and incubated in a cell incubator for 3 h. Finally, the absorbance was detected at 450 nm by a micro-plate reader (Bio-Teck, USA). Each group included three parallels and was repeated three times.

### Colony formation assay

To evaluate the colony-forming ability, the cells were harvested and seeded in 96-well plates (50 cells/well). The number of colonies was counted under an inverted micro-scope on day 7. Each group had three repeats and the experiment was repeated three times.

### Western blot

To detect the protein expression, the western blotting assay was performed. The primary antibodies were used at a dilution of 1:1000 and incubated overnight at 4 °C. The anti-TRIM21 antibody was purchased from Abcam (USA) and the anti-β-Actin antibody was purchased from Sangon Biotech (China). Other antibodies were purchased from Cell Signaling Technology (USA). Goat anti-rabbit or mouse IgG-HRP was used as a secondary antibody at 1:5000 dilution. Detection was performed by using ECL western blotting substrate.

### Co-Immunoprecipitation

Cells were collected and washed after incubated with or without Ab@Tf-Cou6-PLGA NPs for 12 h (200 μg/ml). Co-Immunoprecipitation (Co-IP) experiments were performed with mouse anti-BCR/ABL antibody (Santa, USA), mouse anti-TRIM21 antibody (Santa, USA) and Protein A/G Magnetic beads (MCE, USA). Proteins were detected by rabbit anti-BCR/ABL antibody (Cell Signaling Technology, USA) and rabbit anti-TRIM21 antibody (Abcam, USA).

### Fluorescence imaging

Cells were washed and spread on slides. The cells were fixed with paraformaldehyde, permeabilized with Triton X-100, blocked in goat serum. Afterward, the cells were incubated with primary antibody overnight at 4 °C, and incubated with Cy3- or FITC-labeled secondary antibody (Introvigen, USA). Finally, the nucleus was stained with DAPI (4,6-Diamidino-2-phenylindole, dihydrochloride).

### TRIM21 knock down

To decrease the expression of TRIM21 in CML cells, 50 pmol of siTRIM21 or Control siRNA was transfected into K562 and K562/G01 cells using Cell Line Nucleofector Kit V (Lonza, CHE) according to the instruction. After 24 h, the nanoparticles were added in the culture at a dose of 100 μg/ml, and the cells were harvested after 48 h incubation.

### Clinical information of samples

Human Bone marrow was kindly provided by the Second Affiliated Hospital of Chongqing Medical University. Four samples were collected from CML patients, and three samples were from anemia patients as BCR/ABL negative control (Additional file 7: Table S1). The study was approved by the Ethics Committee of Chongqing Medical University.

### Murine leukemogenesis model

Female NOD/SCID mice (6–7 weeks old) were selected for this study. Before injection, the mice received X-ray radiation at a dose of 250 cGy. Then, each mouse was injected with 5 × 10^6^ K562/G01 cells in 200 μl PBS through the tail vein. The mice were divided into four groups (n = 5, each), the Negative control, PLGA NPs, Tf-Cou6-PLGA NPs, and Ab@Tf-Cou6-PLGA NPs, and were intravenously injected with PBS, PLGA NPs, Tf-Cou6-PLGA NPs, and Ab@Tf-Cou6-PLGA NPs (200 μl, 10 mg/ml) for three times post 1 week of xenograft leukemogenesis. Body weight of the mice was monitored weekly and the white blood cells (WBCs) in the peripheral blood were counted. Bone marrow was harvested from the mice, stained with PE-labeled anti-CD45^+^ human antibody, and the proportion of CD45^+^ cells was detected by FCM. Animal experiments in mice were approved by the Biomedical Ethics Committee of Chongqing Medical University.

### The biosafety and distribution of NPs in vivo

Blood samples of mice were collected post 3 days of NPs intravenous injection. The blood was centrifugated and the serum was separated for biochemical index analysis, including alanine aminotransferase (ALT), aspartate aminotransferase (AST), and blood urea nitrogen (BUN). The main organs of the mice were severed and examined by hematoxylin–eosin (HE) staining. To testify to the distribution of NPs in vivo, the leukemogenesis mice were intravenously injected with Ab@Tf-Cou6-PLGA NPs or Ab@Cou6-PLGA NPs (200 μl, 10 mg/ml). After 24 h of injection, the mice were sacrificed, and the organs and bones were divided for fluorescence imaging analysis (excitation, 475 nm).

### Statistical methods

Statistical analysis was carried out using GraphPad Prism 8.0 software. All data was expressed as a mean ± SD. The statistical significance between groups was assessed by one-way ANOVA. A *p* value < 0.05 was regarded as statistically significant.

## Results

### Synthesis and characteristics of nanoparticles

**Fig. 1:**
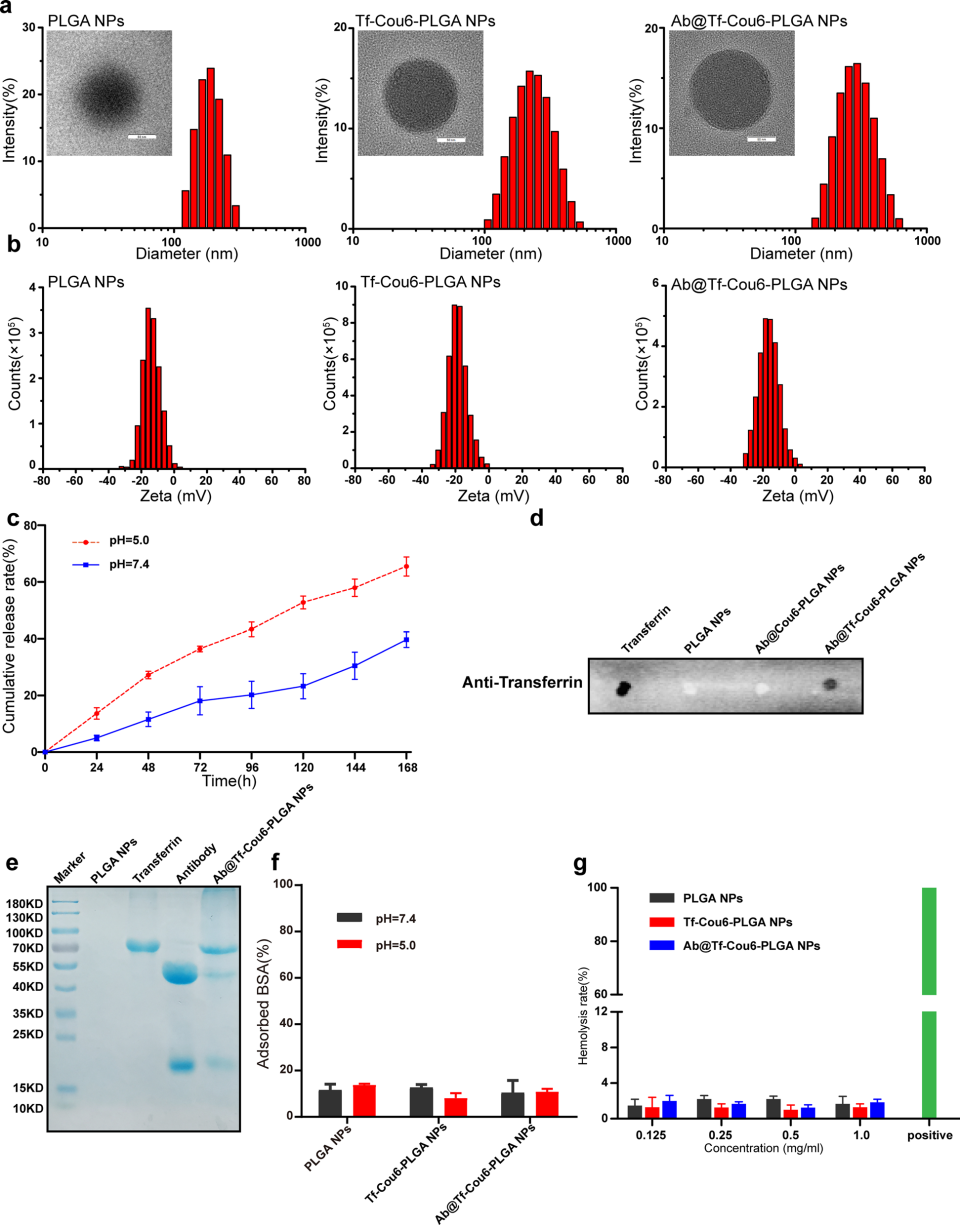
Synthesis and characteristics of nanoparticles. **a** Diameter and TEM images of PLGA NPs, Tf-Cou6-PLGA NPs, and Ab@Tf-Cou6-PLGA NPs. Scale bar, 50 nm. **b** Zeta potential of PLGA NPs, Tf-Cou6-PLGA NPs, and Ab@Tf-Cou6-PLGA NPs. **c** The release rate of Ab@Cou6-PLGA NPs at pH 5.0 and pH 7.4. **d** Dot blotting assay of nanoparticles. **e** Gel electrophoresis analysis of nanoparticles. **f** Protein adsorption rate of nanoparticles at pH 5.0 and pH 7.4. **g** Hemolysis rate of nanoparticles at various concentrations

PLGA NPs were synthesized by the double emulsion solvent evaporation method (Additional file 1: Fig. S1) [40]. Antibodies were encapsulated in the nanoparticles and Cou6 was added in the nanoparticles as a fluorescence probe, and the surface of nanoparticles was modified by transferrin. The characteristics of nanoparticles were measured by TEM and DLS. The result of TEM indicated that the nanoparticles were homogeneous and spherical (Fig. 1a). The diameter and zeta potential of nanoparticles were detected by DLS analysis. As shown in Fig. 1a, b, the diameter of blank PLGA NPs was about 182.50 ± 1.22 nm, and the diameter of Tf-Cou6-PLGA NPs was much larger than the blank nanoparticles at 220.73 ± 1.02 nm. The diameter of Ab@Tf-Cou6-PLGA NPs was about 296.40 ± 5.96 nm. The zeta potential of bank PLGA NPs and Ab@Tf-Cou6-PLGA NPs presented a similar potential (-13.77 ± 0.55 mV to -12.90 ± 0.30 mV), and the zeta potential of Tf-Cou6-PLGA NPs was about -18.73 ± 0.06 mV. Moreover, all nanoparticles exhibited a narrow polydispersity index (PDI), indicating that all the nanoparticles with excellent stability (Additional file 7: Table S2).

To investigate the loading capacity and release rate of antibody in nanoparticles, we synthesized Ab@Cou6-PLGA NPs (Additional file 2: Fig. S2a), and the entrapment efficiency of antibody was about 72.8 ± 2.2% (Additional file 7: Table S3). The release rate of nanoparticles was studied at 37 °C with gentle shaking to simulate the environment in cells, and the results indicated that the release profile was significantly influenced by the pH value. The release speed of antibodies in an acidic environment (60.5 ± 2.3% in 7 days) was faster than it was in neutral (37.2 ± 1.5% in 7 days) (Fig. 1c and Additional file 7: Table S3), indicating that antibodies encapsulated into nanoparticles can be released in the acidic environment of cells. To prove the modification of transferrin on the surface of nanoparticles, the dot blotting assay was carried out, and the result showed the presence of transferrin on Ab@Tf-Cou6-PLGA NPs (Fig. 1d). The protein ingredients of nanoparticles were confirmed by gel electrophoresis analysis. As shown in Fig. 1e, the protein band in Ab@Tf-Cou6-PLGA NPs was similar to the transferrin and antibody, while zero protein was found in PLGA NPs band, illustrating that the antibodies were successfully loaded into the Ab@Tf-Cou6-PLGA NPs (Fig. 1e). The protein electrostatic absorption of nanoparticles causes clearance by the reticuloendothelial system (RES) during blood circulation [41]. In light of this, we tested the protein absorption rate of nanoparticles, and results showed that all nanoparticles, whether at pH 5.0 or pH 7.4, had a weak protein adsorption ability, indicating that nanoparticles with great stability (Fig. 1f). To assess the blood biocompatibility of nanoparticles, the hemolysis assay was performed. As displayed in Fig. 1g, the nanoparticles at the tested concentrations exhibited a low hemolysis rate, indicating that nanoparticles have a great potential for in vivo application.

### The cellular uptake of nanoparticles

**Fig. 2:**
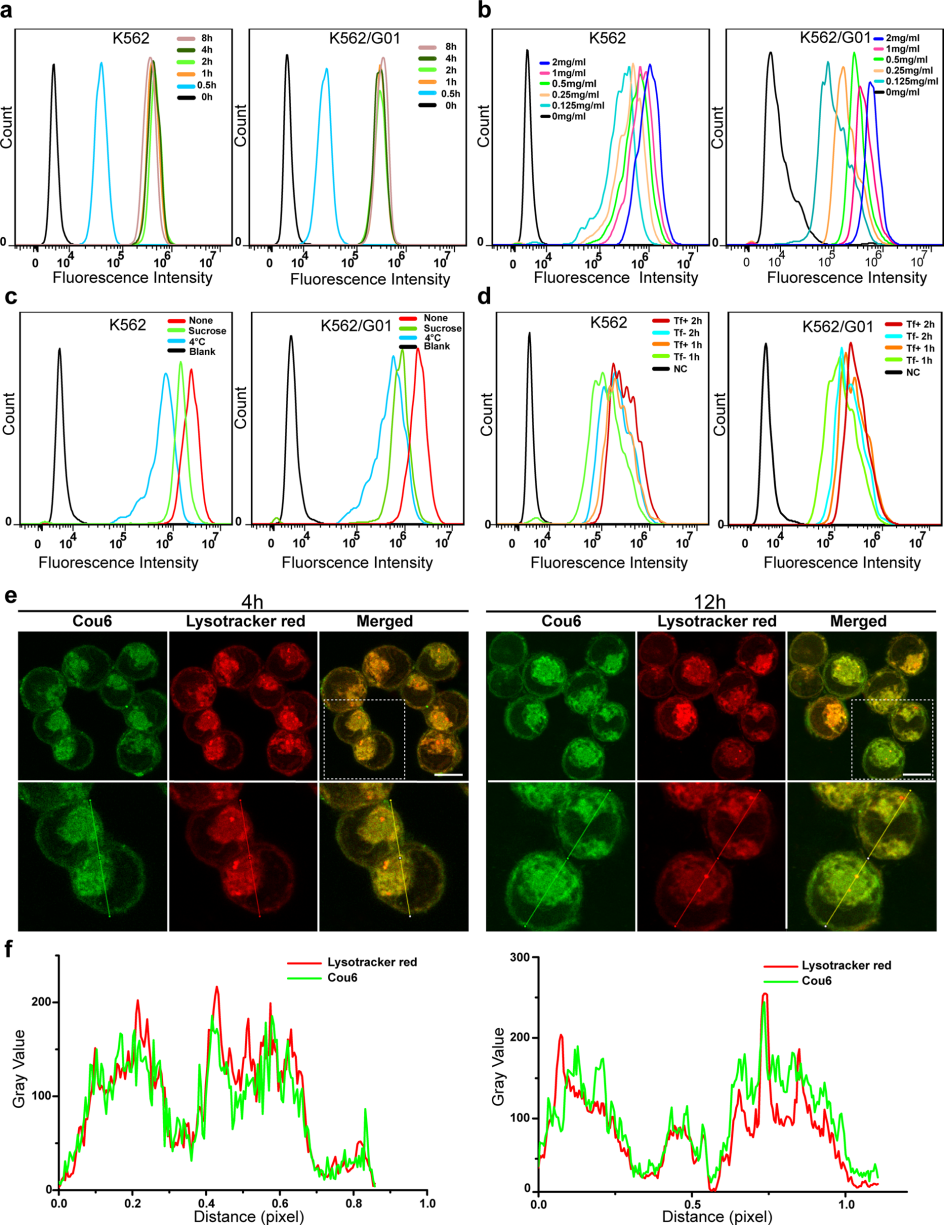
The cellular uptake of nanoparticles. **a** FCM analysis of the intracellular of Ab@Tf-Cou6-PLGA NPs in a time-dependent manner at the dose of 0.5 mg/ml. **b** FCM analysis of intercellular uptake of Ab@Tf-Cou6-PLGA NPs in K562 and K562/G01 cells at various doses. **c** FCM analysis of cellular internalization of Ab@Tf-Cou6-PLGA NPs in K562 and K562/G01 cells after Sucrose (0.45 mM) and 4 °C treatments. **d** FCM analysis of the cellular uptake of Tf-targeted and non-targeted nanoparticles in CML cells. **e** CLSM images of K562 cells after incubation with Ab@Tf-Cou6-PLGA NPs. Scale bar, 10 μm. **f** Colocalization analysis of K562 cells after incubation with Ab@Tf-Cou6-PLGA NPs

The intracellular delivery of nanoparticles in K562 and K562/G01 cells was detected by a fluorescence microscope and FCM. To visualize the intracellular of nanoparticles, Cou6 was added in the nanoparticles for its great green fluorescence intensity. As shown in Fig. 2a and Additional file 3: Fig. S3a, the cellular uptake of nanoparticles showed time dependence, it was started at 0.5 h, and maintained dynamic stability for 1 h to 8 h. Furthermore, the intracellular of nanoparticles showed dose dependence, with the intensity of cell fluorescence increasing as the nanoparticle dose increased (Fig. 2b and Additional file 3: Fig. S3b). The intracellular delivery of nanoparticles was also visually reflected by TEM (Additional file 3: Fig. S3c). To verify the intracellular internalization mechanism of nanoparticles, we further investigated the cellular uptake capacity of nanoparticles after using sucrose to block endocytosis or culturing at 4 °C to reduce cell membrane activity. As displayed in Fig. 2c and Additional file 3: Fig. S3d, cells treated at 4 °C showed blocked nanoparticle internalization. Cells treated with sucrose also showed impactful inhibition of the cellular uptake of nanoparticles. These results suggested that the intracellular uptake of nanoparticles was energy-dependent and clathrin-mediated endocytosis. Subsequently, we found that transferrin modified nanoparticles exhibited better cell internalization than the nanoparticles without transferrin modification, indicating that transferrin modification increased the CML cell uptake of nanoparticles (Fig. 2d and Additional file 3: Fig. S3e). To study the intracellular distribution of nanoparticles, CML cells were stained by lysotracker red after treated with Ab@Tf-Cou6-PLGA NPs. As shown in Fig. 2e, f, after treated with Ab@Tf-Cou6-PLGA NPs for 4 h, the obvious yellow color could be observed in cells, indicating that most nanoparticles were located in the lysosome after 4 h incubation. Next, when cells were further incubated for 12 h, the green fluorescence of Ab@Tf-Cou6-PLGA NPs and the red fluorescence of lysotracker were separated, indicating that Ab@Tf-Cou6-PLGA NPs had escaped from the lysosome.

### The expression of BCR/ABL protein in CML cells was decreased after Ab@Tf-Cou6-PLGA NPs treatment

**Fig. 3:**
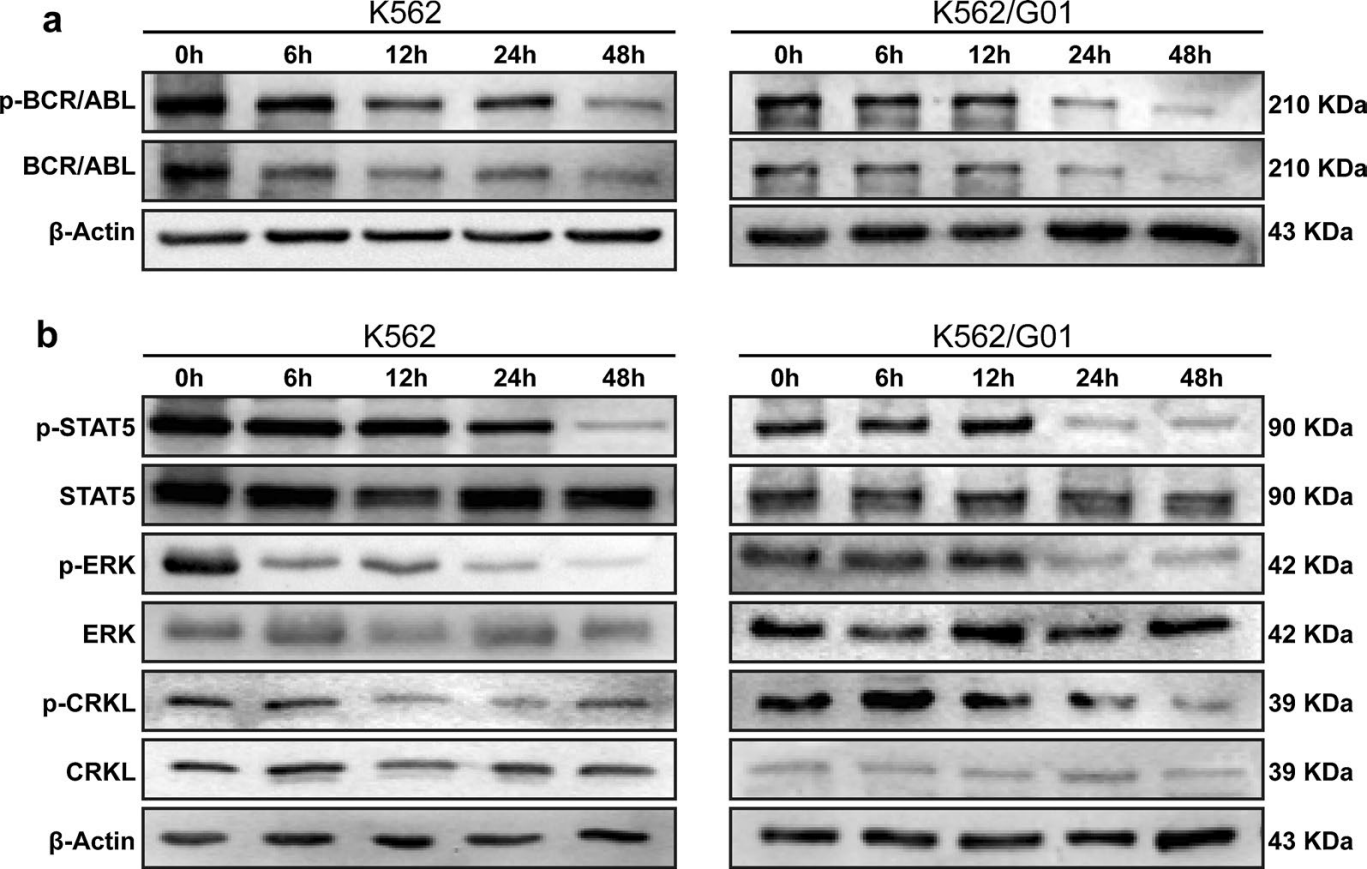
The expression of BCR/ABL protein in CML cells was decreased after Ab@Tf-Cou6-PLGA NPs treatment. **a** The expression of BCR/ABL and p-BCR/ABL in CML cells after Ab@Tf-Cou6-PLGA NPs treatment was detected by western blot assay. **b** The expression of p-STAT5, p-ERK and p-CRKL in CML cells was detected by western blot assay

To testify whether Ab@Tf-Cou6-PLGA NPs treatment promotes BCR/ABL degradation, a western blot assay was performed. The quantity of BCR/ABL and its phosphorylation was gradually diminished over time after the treatment of Ab@Tf-Cou6-PLGA NPs both in K562 and K562/G01 cells (Fig. 3a). Furthermore, we detected the downstream molecules of phosphor-BCR/ABL. Simultaneous decreased expression of phosphor-STAT5, phosphor-ERK, and phosphor-CRKL was observed in the Ab@Tf-Cou6-PLGA NPs treated groups (Fig. 3b). As illustrated in Additional file 4: Fig. S4a, the expression of BCR/ABL and phosphor-BCR/ABL was significantly decreased in the transferrin targeted group compared with the non-targeted Ab@Cou6-PLGA NPs group. Furthermore, the Ab@Tf-Cou6-PLGA NPs could degrade the BCR/ABL protein, while imatinib failed to degrade the BCR/ABL protein in the imatinib resistance cell line K562/G01 (Additional file 4: Fig. S4c). As a group, these results demonstrated that the expression of phosphor-BCR/ABL and its downstream signaling molecules in CML cells were down-regulated after Ab@Tf-Cou6-PLGA NPs treatment.

### Ab@Tf-Cou6-PLGA NPs suppressed proliferation and induced apoptosis of CML cells

**Fig. 4:**
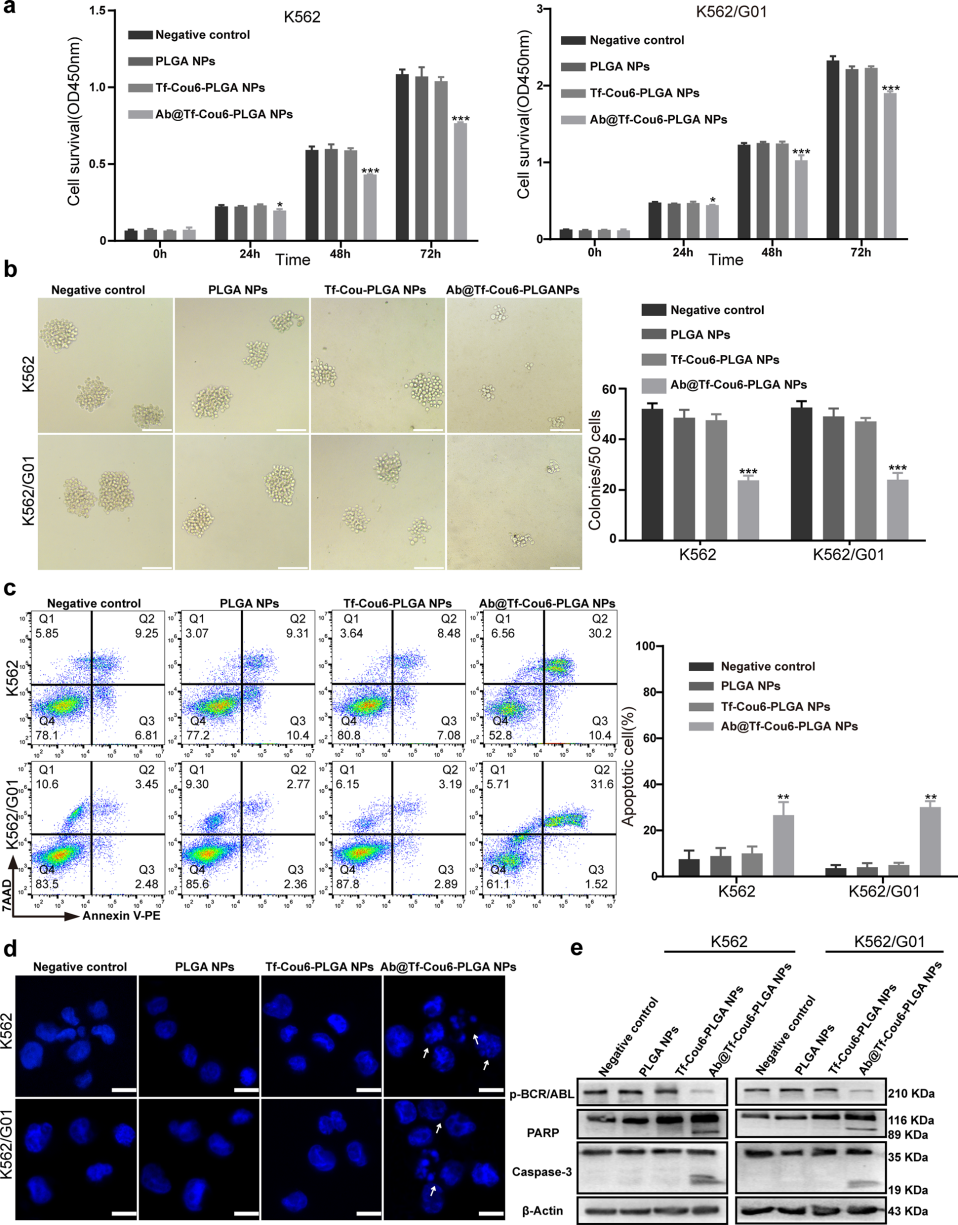
Ab@Tf-Cou6-PLGA NPs suppressed proliferation and induced apoptosis of CML cells. **a** The effect of nanoparticles on CML cells was tested by CCK-8 assay. **b** The number of colonies was counted. Scale bar, 100 μm. **c** The apoptosis rate of CML cells was detected by FCM. **d** Morphologic changes of apoptosis were tasted by DAPI staining. The typical apoptosis cells were pointed out by arrows. Scale bar, 10 μm. **e** The expression of cleaved PARP and cleaved caspase-3 was assessed by western blot assay. Data are presented as the means ± SD. *p < 0.05, **p < 0.01, ***p < 0.001, ****p < 0.0001

To testify to the function of nanoparticles on the proliferation and colony-forming ability of CML cells, the CCK-8 and colony-forming assays were carried out. As illustrated in Fig. 4a, the proliferation ability of Ab@Tf-Cou6-PLGA NPs treated cells was significantly suppressed compared with other groups. Meanwhile, no statistical differential in cell proliferation ability was observed among control groups. Furthermore, to identify whether Ab@Tf-Cou6-PLGA NPs have cytotoxicity, the same experiment was performed in BCR/ABL negative cells, including HL-60, NB4, and A549 cells. The results demonstrated that there was no significant difference in cell proliferation ability among all groups (Additional file 4: Fig. S4d). The results of the colony-forming assay indicated that the colony-forming ability of cells was significantly suppressed by Ab@Tf-Cou6-PLGA NPs. Contrarily, other treatments showed no effect on the colony-forming ability of cells (Fig. 4b).

Therefore, to test the influence of nanoparticles on the apoptosis of CML cells, K562 and K562/G01 cells were incubated with nanoparticles. Apoptosis of cells was detected by FCM. The results demonstrated that the apoptosis rate of CML cells was significantly increased in the Ab@Tf-Cou6-PLGA NPs treated group compared with other groups (Fig. 4c). The cells in the transferrin modified Ab@Tf-Cou6-PLGA NPs treated group showed a higher apoptosis rate compared with the Ab@Cou6-PLGA NPs group (Additional file 4: Fig. S4b). To investigate the influence of NPs on BCR/ABL negative cells, the apoptosis rate of A549, HL-60, and NB4 was detected by the FCM. As the results showed, the NPs had no apparent influence on the apoptosis of BCR/ABL negative cells (Additional file 4: Fig. S4e). Furthermore, DAPI staining was performed to visualize the nuclear morphology changes. As displayed in Fig. 4d, morphological changes of apoptosis were increased in the Ab@Tf-Cou6-PLGA NPs treated group. In contrast, no apparent morphological changes were observed in other groups. Besides, a western blot assay was carried out and results showed that the expression of cleaved PARP and cleaved caspase-3 was dramatically increased both in K562 and K562/G01 cells after treatment by Ab@Tf-Cou6-PLGA NPs (Fig. 4e). To sum up, Ab@Tf-Cou6-PLGA NPs treatment suppressed proliferation and promoted apoptosis of both imatinib sensitive and resistant CML cells.

### The role of the Trim-Away pathway in Ab@Tf-Cou6-PLGA NPs-mediated BCR/ABL degradation

**Fig. 5:**
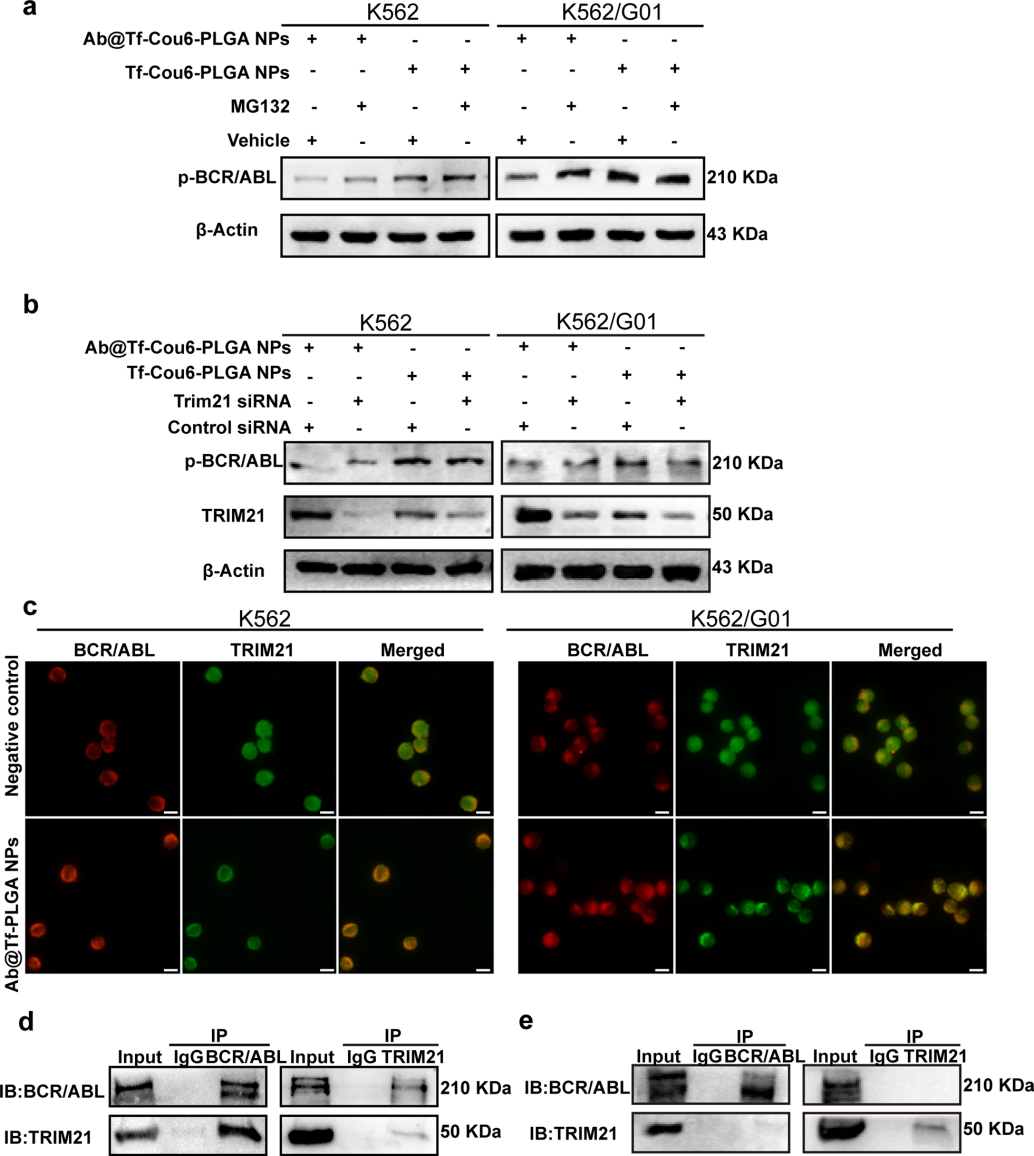
The role of Trim-Away pathway in Ab@Tf-Cou6-PLGA NPs-mediated BCR/ABL degradation. **a** Proteasome inhibitor MG132 (100 nM) treatment weakened the BCR/ABL degradation effects of Ab@Tf-Cou6-PLGA NPs in CML cells. **b** The suppression effect of Ab@Tf-Cou6-PLGA NPs on BCR/ABL was reduced by TRIM21 knockdown. **c** Immunofluorescence assay was used to analyze the colocalization of BCR/ABL and TRIM21 after Ab@Tf-PLGA NPs treatment. Scale bar, 10 μm. **d** Co-IP analysis of the interaction of BCR/ABL and TRIM21 with Ab@Tf-Cou6-PLGA NPs treatment. **e** Co-IP analysis of the interaction of BCR/ABL and TRIM21 without Ab@Tf-Cou6-PLGA NPs treatment

Previous studies revealed that intracellular antibodies could specifically degrade endogenous protein via the Trim-Away pathway [18]. Consequently, we explored the role of TRIM21 and proteasomes in Ab@Tf-Cou6-PLGA NPs-induced BCR/ABL degradation. To testify to the role of proteasomes in the Trim-Away pathway, the proteasome inhibitor MG132 was employed. As displayed in Fig. 5a, compared with other groups, the addition of MG132 prevented the p-BCR/ABL degradation which was induced by Ab@Tf-Cou6-PLGA NPs treatment. To further confirm the function of TRIM21 in Ab@Tf-Cou6-PLGA NPs-mediated BCR/ABL degradation, CML cells were transfected with siTRIM21 or non-target siRNA. As expected, the degradation of the BCR/ABL protein mediated by Ab@Tf-Cou6-PLGA NPs was reduced in siTRIM21 transfected cells compared with cells in the control group (Fig. 5b). These results supported the idea that the p-BCR/ABL protein was degraded by Ab@Tf-Cou6-PLGA NPs via the Trim-Away pathway. To confirm the colocalization of BCR/ABL and TRIM21 in CML cells, Ab@Tf-PLGA NPs were synthesized to eliminate the green fluorescence interference of Cou6 (Additional file 2: Fig. S2b; Additional file 7: Table S2). As the results showed in Fig. 5c, there was a higher colocalization of BCR/ABL and TRIM21 in Ab@Tf-PLGA NPs treated cells compared with the control group, indicating an association between BCR/ABL and TRIM21 after Ab@Tf-PLGA NPs treatment. To certify whether the interaction of TRIM21 and BCR/ABL was mediated by Ab@Tf-Cou6-PLGA NPs, cells were incubated with or without Ab@Tf-Cou6-PLGA NPs. The Co-IP assay revealed that BCR/ABL interacts with TRIM21 in the presence of Ab@Tf-Cou6-PLGA NPs (Fig. 5d), while there was no interaction between BCR/ABL and TRIM21 in the control group (Fig. 5e). These results suggested that Ab@Tf-Cou6-PLGA NPs induced BCR/ABL degradation via the Trim-Away pathway.

### The apoptosis was induced by Ab@Tf-Cou6-PLGA NPs in cells from CML patients

To confirm whether Ab@Tf-Cou6-PLGA NPs worked in CML patient cells, cells from CML patients were co-cultured with NPs. The apoptosis rate of cells was determined by FCM. The results indicated that cells co-cultured with Ab@Tf-Cou6-PLGA NPs showed a higher apoptosis rate compared with other groups (Additional file 5: Fig. S5a, 5c). In contrast, the NPs showed no significant effect on the cells extracted from anemia patients (Additional file 5: Fig. S5b, 5d). These results illustrated that Ab@Tf-Cou6-PLGA NPs increased the apoptosis rate of cells from CML patients, and no significant effects were observed on cells from BCR/ABL negative donors.

### The oncogenesis of CML cells was impaired by Ab@Tf-Cou6-PLGA NPs in vivo

**Fig. 6:**
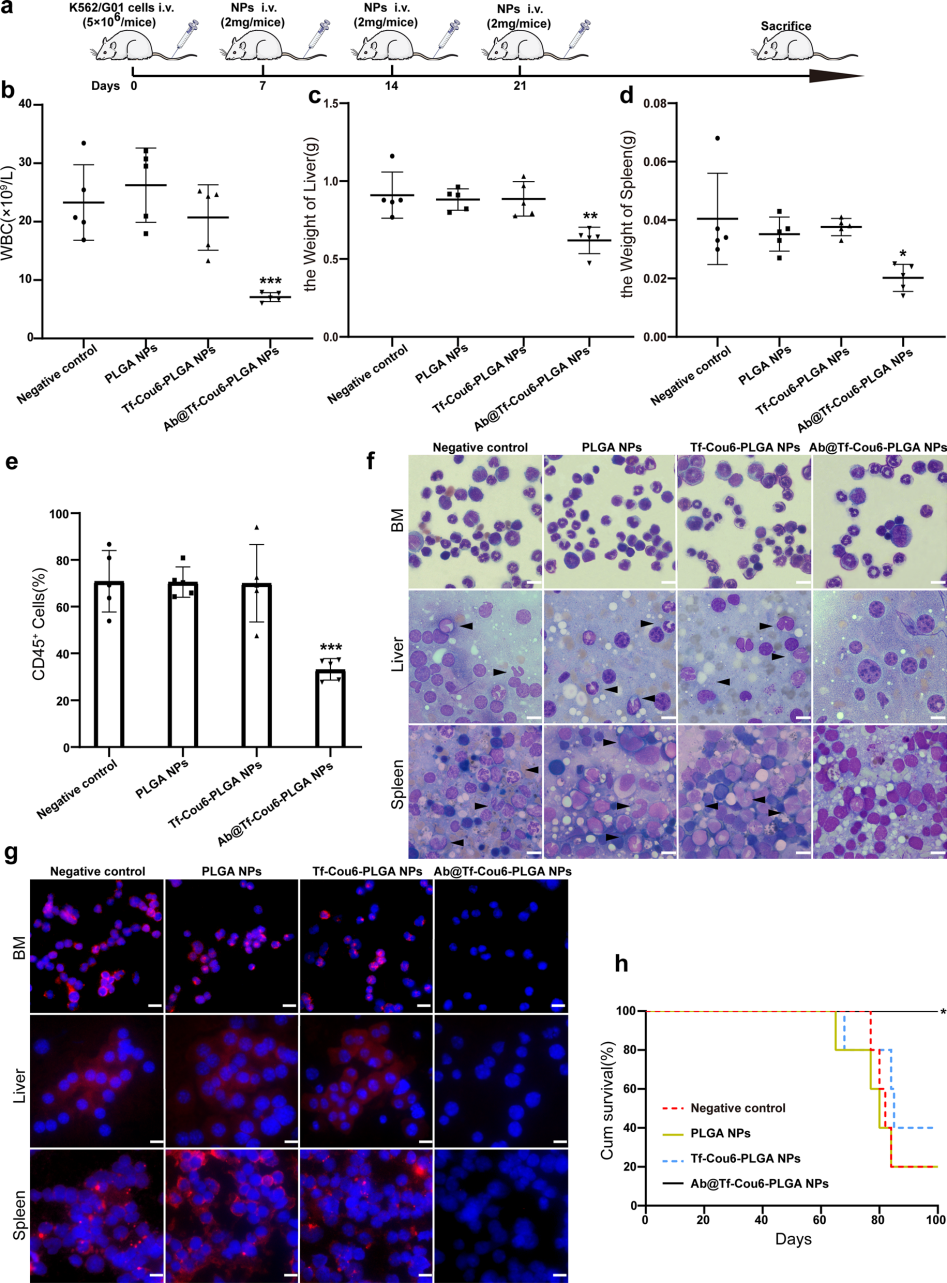
The oncogenesis of CML cells was impaired by Ab@Tf-Cou6-PLGA NPs in vivo. **a** Treatment schedule for CML mice model. **b** The maximum WBC counts of mice were recorded. **c**, **d** The weights of the liver and spleen of mice were measured. **e** The percent of CD45^+^ cells in the bone marrow of mice were detected by FCM. **f** Cells from bone marrow, liver, and spleen of mice were tested by Wright’s stain. The arrows indicate leukemic cells. Scale bar, 10 μm. **g** The expression of BCR/ABL was observed by immunofluorescence test. Scale bar, 10 μm. **h** The survival curves of mice were analyzed by Kaplan–Meier methods. Data are presented as the means ± SD. *p < 0.05, **p < 0.01, ***p < 0.001, ****p < 0.0001

To investigate whether Ab@Tf-Cou6-PLGA NPs treatment could exert anti-leukemia efficacy in vivo, we established a CML model in immunodeficient NOD/SCID mice. The mice received X-ray radiation at a dose of 250 cGy before being injected with 5 × 10^6^ K562/G01 cells. The mice received different NPs at a dosage of 2 mg/week or PBS control treatment for three weeks through the tail vein at 1-week post intravenous injection with K562/G01 cells (Fig. 6a). The weight and WBC count of the mice were monitored weekly. As illustrated in Fig. 6b, the WBC peak level of each mouse in the Ab@Tf-Cou6-PLGA NPs treated group was dramatically lower than in other groups. Furthermore, the liver and spleen of mice were weighted, and we noticed that the mice in the negative control, PLGA NPs, and Tf-Cou6-PLGA NPs groups developed more severe hepatosplenomegaly compared with the Ab@Tf-Cou6-PLGA NPs group (Fig. 6c, d and Additional file 6: Fig. S6a). To evaluate leukemia progression, the bone marrow of mice was harvested. The proportion of human-CD45 positive cells was detected by FCM. The results confirmed that the Ab@Tf-Cou6-PLGA NPs treated group had a significantly lower proportion of CD45-positive cells compared with other groups (Fig. 6e). Besides, all mice were weighed before CML cells injection, and the final weight of each mouse was recorded. As expected, the body weight of mice in the negative control, PLGA NPs, and Tf-Cou6-PLGA NPs groups was significantly decreased. Inversely, the body weight of mice in the Ab@Tf-Cou6-PLGA NPs treated group only showed a slight reduction (Additional file 6: Fig. S6b). The tissue infiltration of CML cells was examined by HE staining and Wright’s staining. The results revealed that there was a mild leukemic cell infiltration from the Ab@Tf-Cou6-PLGA NPs treated group compared with the negative control, PLGA NPs and Tf-Cou6-PLGA NPs (Fig. 6f and Additional file 6: Fig. S6c). The results of Wright’s staining of the bone marrow showed that the Ab@Tf-Cou6-PLGA NPs treated group exhibited less infiltration of leukemic cells than other groups (Fig. 6f). In addition, the results of the immunofluorescent assay revealed that there was a less BCR/ABL protein expression in the liver, spleen, and bone marrow of the Ab@Tf-Cou6-PLGA NPs treated group than in other groups (Fig. 6g). Moreover, Kaplan–Meier survival curves demonstrated that the survival time of mice in the Ab@Tf-Cou6-PLGA NPs treated group was longer than in other groups (Fig. 6h). Taken together, these results supported that the Ab@Tf-Cou6-PLGA NPs treatment impaired the oncogenesis capability of K562/G01 cells in mice.

### Biosafety and distribution of nanoparticles in vivo

**Fig. 7:**
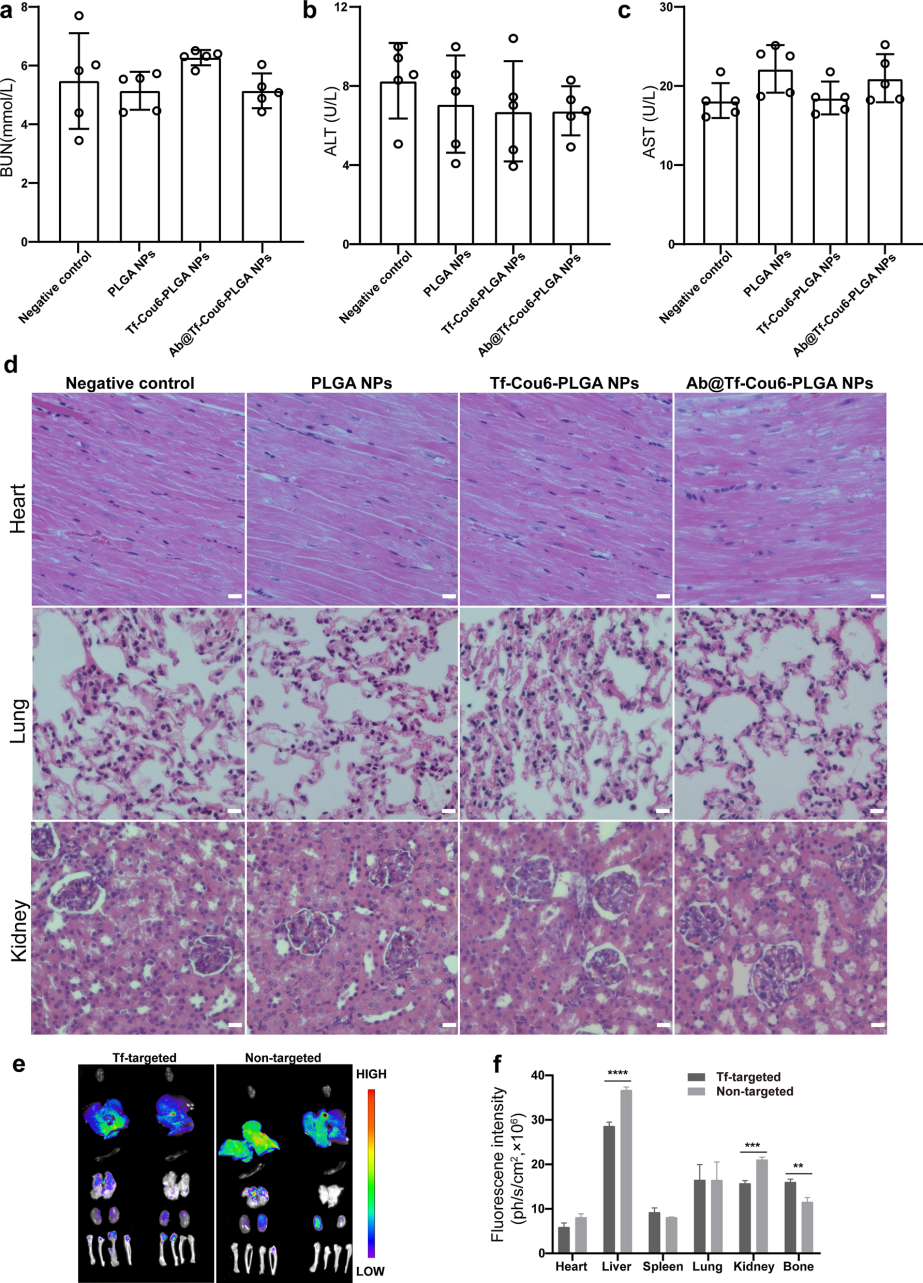
Biosafety and distribution of nanoparticles in vivo. **a**–**c** The BUN, ALT, and AST index in peripheral blood were detected. **d** HE staining for major organs of mice. Scale bar, 10 μm. **e** Fluorescence images of major organs and bones anatomized from mice 24 h post intravenous injection of transferrin targeted Ab@Tf-Cou6-PLGA NPs and non-targeted Ab@Cou6-PLGA NPs

To testify to the biocompatibility of NPs, serum samples from CML model mice were collected 3 days after NPs treatment. The blood indexes, including AST, ALT for liver function, and BUN for kidney function, were measured. As shown in Fig. 7a–c, there was no statistical significance in AST, ALT, and BUN between the PBS control and NPs treated groups, indicating that the NPs had no hepatic and renal toxicity in vivo. Furthermore, parallel to the negative control group, no tissue injury or inflammatory lesions was detected in the NPs treated groups (Fig. 7d). Above all, PLGA NPs, Tf-Cou6-PLGA NPs, and Ab@Tf-Cou6-PLGA NPs showed excellent biocompatibility in mice.

To investigate the targeting ability of Ab@Tf-Cou6-PLGA NPs in vivo, transferrin-modified Ab@Tf-Cou6-PLGA NPs and unmodified Ab@Cou6-PLGA NPs were injected into CML model mice at a dose of 2 mg through the tail vein. The mice were sacrificed after 24 h of intravenous injection, and their major organs, including the heart, liver, spleen, lung, kidney, and bone, were subjected to fluorescence imaging. The results in Fig. 7e, f showed an apparent difference in fluorescence intensity in bone marrow. The intensity in the transferrin labeled group was higher than in the non-targeted group, indicating that modification with transferrin achieved higher delivery and protein targeting in CML cells.

## Discussion

CML is a myeloproliferative disorder derived from the reciprocal translocation t (9, 22) (q34; q11), which creates the bcr/abl fusion gene. The BCR/ABL oncoprotein, which is encoded by the bcr/abl fusion gene, possesses abnormal kinase activity and induces cell malignancy [3]. As the BCR/ABL protein plays an essential role in promoting CML cell leukemogenesis, inhibiting the tyrosine kinase activity of BCR/ABL or eliminating BCR/ABL is the first choice for treatment of CML. The development of TKI has revolutionized CML therapy. TKI can compete with ATP or substrate, specifically inhibit the tyrosine kinase activity of BCR/ABL, and imatinib is now the first-line therapy for chronic-phase CML [42]. TKI use, on the other hand, raises the issue of drug intolerance or resistance [9]. Mutations of the BCR/ABL kinase region change the conformation of BCR/ABL, which affects its combination with Imatinib. The emergence of the second generation TKI is effective for some mutations, but patients with T315I mutation are widely resistant to the first generation TKI (imatinib) and the second generation TKI (nilotinib, dasatinib). The third generation TKI is effective for the patients with T315I mutation, but its effectiveness comes at the cost of higher grade 3–4 toxicity and more serious adverse drug events, such as cardiovascular events and pleural effusion [5]. Therefore, a new method is urged for CML treatment.

**Fig. 8:**
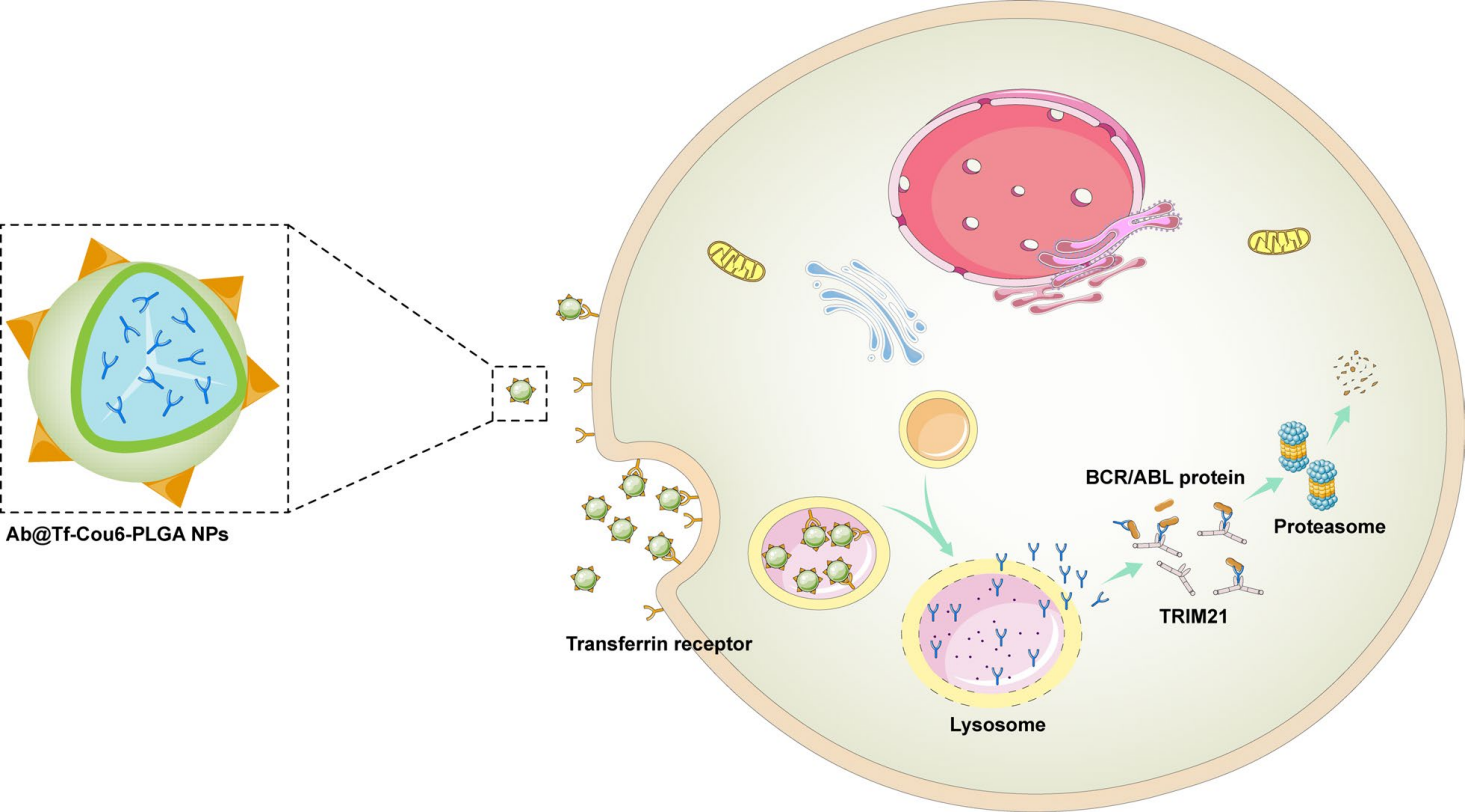
Schematic diagram of degraded the BCR/ABL oncoprotein by Ab@Tf-Cou6-PLGA NPs. The anti-BCR/ABL antibodies were loaded into nanoparticles, and the transferrin was modified on the surface of Ab@Cou6-PLGA NPs to increase the CML targeting ability. The Ab@Tf-Cou6-PLGA NPs could deliver anti-BCR/ABL antibodies into CML cells. In the acidic environment of the lysosome of CML cells, the Ab@Tf-Cou6-PLGA NPs were degraded and the anti-BCR/ABL antibodies were released. The anti-BCR/ABL antibodies can specifically bind to the BCR/ABL oncoprotein and degrade the BCR/ABL protein via the Trim-Away pathway

In this study, we provided a new strategy based on the Trim-Away pathway and a nanoparticle intracellular delivery system to specifically target the BCR/ABL oncoprotein (Fig. 8). In our study, we achieved anti-BCR/ABL antibody intracellular delivery and degrading the BCR/ABL oncoprotein via the Trim-Away pathway, eventually eliminating the pathogenicity of CML cells. Because most antibodies are unable to cross the cell membrane, their intracellular application is limited [43, 44]. To solve this problem, we employed PLGA nanoparticles as an antibody intracellular delivery carrier owing to their high maneuverability, good biosafety, and strong drug loading capability [45]. Previous studies have reported that antibodies can be transferred into cells by cell transduction peptide or virus vector to act in combination with pathogenic proteins [46, 47]. Compared to other antibody intracellular delivery methods such as cell-penetrating peptides and viral vectors, PLGA NPs can protect antibodies from elimination in blood circulation, with great biocompatibility, and can be easily modified with targeting. Furthermore, the synthesis process can be easily scaled up for mass production of nanoparticles. To increase the CML cell targeting of nanoparticles, transferrin was labeled on the surface of PLGA NPs, on account of a higher expression level in CML cells compared with normal cells [48]. In our study, we confirmed that the modification of transferrin enhanced the intracellular ability of NPs, and the fluorescence imaging of the mice’s organs and bones revealed that the modification of transferrin on nanoparticles increased the CML cells’ targeting (Figs. 2d, 7e, f). Compared to traditional drugs [49, 50], we transported specific antibodies into cells with less toxic effects. The results of BUN, ALT, AST index in peripheral blood, and HE staining of tissue indicated all nanoparticles have excellent biocompatibility in mice (Fig. 7).

In our study, we proved that Ab@Tf-Cou6-PLGA NPs treatment can effectively suppress the proliferation and promote apoptosis of both Imatinib sensitive and resistant CML cells, illustrating that Ab@Tf-Cou6-PLGA NPs achieved excellent anti-oncogenesis efficacy in vitro (Fig. 4). The efficacy of NPs in the CML leukemic model was proven, and the results suggested that the Ab@Tf-Cou6-PLGA NPs can suppress CML leukemogenesis in vivo (Fig. 6). These results suggest that degrading BCR/ABL oncoprotein by Ab@Tf-Cou6-PLGA NPs is a promising clinical tool for CML therapy. Unlike the TKIs treatment, the intracellular anti-BCR/ABL antibody can directly degrade the BCR/ABL oncoprotein via the Trim-Away pathway and achieve an equal treatment effect both in TKI sensitive and resistant CML cells regardless of all mutations. Compared to knocking out the bcr/abl pathogenic gene with the CRISPER techniques, degrading the BCR/ABL oncoprotein by intracellular antibodies showed excellent biosafety, with no off-target risk. Compared to the adoptive immune therapy, degrading BCR/ABL oncoprotein by Ab@Tf-Cou6-PLGA NPs is simple to operate, since adoptive immune therapy needs to activate anticancer immune cells in vitro in advance and transfuse these cells back to patients, Ab@Tf-Cou6-PLGA NPs have better biosafety and no adverse reactions such as cytokine storms. While it was reported that the PROTAC technology can specifically degrade BCR/ABL protein by ubquitin-proteasome system, the bioavailability and cell penetration of PROTACs need further improvement due to large molecular weight of PROTACs. As PROTACs belong to small molecule compound, toxicity is always a matter of concern. According to this, degrading the BCR/ABL oncoprotein by intracellular antibodies has a better bioavailability and biosafety in comparison.

Given the experimental results presented here, degrading the BCR/ABL protein by intracellular antibodies via the Trim-Away pathway may be a potential candidate for CML therapy. However, the transferrin is not a CML specific target, cell-specific targeting of nanoparticles needs to be further developed. As previous studies reported, CD26 and interleukin-1 receptor associated protein (IL1RAP) were targets for CML stem cells [51, 52]. Depending on this, anti-CD26 antibody or anti-IL-1RAP antibody can be modified on the surface of nanoparticles to achieve cell-specific targeting to CML stem cells in future studies.

In summary, this is the first report of using intracellular delivery of antibodies by nanoparticles for degrading BCR/ABL oncoprotein via the Trim-Away pathway. In our study, we demonstrated that intracellular delivery of anti-BCR/ABL antibodies by PLGA NPs exhibited an excellent anti-oncogenesis ability for CML in vitro and in vivo. The anti-BCR/ABL antibodies intracellular delivery by transferrin-modified PLGA NPs achieved curative effects and provided a potential therapy for CML.

## Conclusions

In this study, we successfully synthesized Ab@Tf-Cou6-PLGA NPs for the intracellular delivery of anti-BCR/ABL antibodies. We demonstrated that Ab@Tf-Cou6-PLGA NPs could specifically degrade BCR/ABL protein via the Trim-Away pathway and suppress the pathogenesis of CML cells in vivo and in vitro. This method provides a promising therapeutic strategy for CML patients, particularly for TKI resistance patients.

## Supplementary Information

**Additional file 1: Fig. S1.** Schematic of the double emulsion solvent evaporation method to formulate Ab@Tf-Cou6-PLGA NPs. The anti-BCR/ABL antibodies were mixed with chloroform containing PLGA and Cou6. The mixture was sonicated to obtain the first emulsion. Then, the first emulsion was mixed with 10 ml of PVA (1%) and sonicated to obtain the second emulsion. Furthermore, the chloroform was evaporated at room temperature to obtain the Ab@Cou6-PLGA NPs. The Ab@Tf-Cou6-PLGA NPs were synthesized after being modified with transferrin.

**Additional file 2: Fig. S2.** Synthesis and characteristics of nanoparticles. (a) Diameter and Zeta potential of Ab@Cou6-PLGA NPs. (b) Diameter and Zeta potential of Ab@Tf-PLGA NPs.

**Additional file 3: Fig. S3.** The cellular uptake of nanoparticles. (a) Fluorescence images of Ab@Tf-Cou6-PLGA NPs uptake into CML cells in a time-dependent manner. Scale bar, 10 μm. (b) Fluorescence images of intercellular uptake of Ab@Tf-Cou6-PLGA NPs in K562 and K562/G01 cells at various doses. Scale bar, 10 μm. (c) TEM images of cellular uptake of nanoparticles. The blank arrows indicate nanoparticles. Scale bar, 500 nm. (d) Fluorescence images of cellular internalization of Ab@Tf-Cou6-PLGA NPs in K562 and K562/G01 cells after Sucrose (0.45 mM) and 4 °C treatment. Scale bar, 10 μm. (e) Fluorescence images of cellular uptake of Tf-targeted and non-targeted nanoparticles in K562 cells. Scale bar, 10 μm. (f) Fluorescence images of cellular uptake of Tf-targeted and non-targeted nanoparticles in K562/G01 cells. Scale bar, 10 μm.

**Additional file 4: Fig. S4.** Expression of BCR/ABL oncoprotein in nanoparticles treated CML cells. (a) The BCR/ABL and p-BCR/ABL expression level in Tf-targeted or non-targeted nanoparticles treated CML cells. (b) The apoptosis rate of CML cells after treated for 48h by Ab@Cou6-PLGA NPs or Ab@Tf-Cou6-PLGA NPs was detected by FCM. (c) The BCR/ABL expression level in CML cells after being treated by Ab@Tf-Cou6-PLGA NPs or imatinib. (d) The effect of nanoparticles on BCR/ABL negative cells was detected by CCK-8. (e) The apoptosis rate of BCR/ABL negative cells after being treated for 48h by nanoparticles was detected by FCM.

**Additional file 5: Fig. S5.** The apoptosis was induced by Ab@Tf-Cou6-PLGA NPs in cells from CML patients. (a, c) The apoptosis rate of cells from CML patients was tested by FCM. (b, d) The apoptosis rate of cells from BCR/ABL negative donors was tested by FCM. Data are presented as the means ± SD. *P < 0.05, **P < 0.01, ***p < 0.001, ****p < 0.0001.

**Additional file 6: Fig. S6.** The oncogenesis of CML cells in vivo was impaired by Ab@Tf-Cou6-PLGA NPs. (a) Images of livers and spleens form each group. (b) The initial weight and final weight of mice were recorded of each mouse. (c) The infiltration leukemic cells in the spleens and livers were analyzed by HE staining. The black arrows indicate leukemic cells. The black arrows indicate leukemic cells. Scale bar, 10 μm. Data are presented as the means ± SD. *P < 0.05, **P < 0.01, ***p < 0.001, ****p < 0.0001.

**Additional file 7: Supplement tables.Table S1**. Patient’s information.** Table S2**. Nanoparticles and their properties.** Table S3**. Entrapment efficiency and release rate of nanoparticles.

## Data Availability

Not applicable.

## Abbreviations

CML: Chronic myeloid leukemia
TKIs: Tyrosine kinase inhibitors
PLGA: Poly(d, l-lactide-*co*-glycolide)
FCM: Flow cytometry
DLS: Dynamic light scattering
TEM: Transmission electron microscopy
HSCT: Allogeneic bone marrow transplantation
TRIM21: Tripartite motif containing 21
Tf: Transferrin
WBCs: White blood cells
HE: Hematoxylin-eosin

## References

[1] Bartram C, de Klein A, Hagemeijer A, van Agthoven T, Geurts van Kessel A, Bootsma D, Grosveld G, Ferguson-Smith M, Davies T, Stone M (1983). Translocation of c-ab1 oncogene correlates with the presence of a Philadelphia chromosome in chronic myelocytic leukaemia. Nature.

[2] Soverini S, Mancini M, Bavaro L, Cavo M, Martinelli G (2018). Chronic myeloid leukemia: the paradigm of targeting oncogenic tyrosine kinase signaling and counteracting resistance for successful cancer therapy. Mol Cancer.

[3] Ben-Neriah Y, Daley G, Mes-Masson A, Witte O, Baltimore D (1986). The chronic myelogenous leukemia-specific P210 protein is the product of the bcr/abl hybrid gene. Science (New York, NY).

[4] Ren R (2005). Mechanisms of BCR-ABL in the pathogenesis of chronic myelogenous leukaemia. Nat Rev Cancer.

[5] Braun T, Eide C, Druker B (2020). Response and resistance to BCR-ABL1-targeted therapies. Cancer Cell.

[6] Rosti G, Castagnetti F, Gugliotta G, Baccarani M (2017). Tyrosine kinase inhibitors in chronic myeloid leukaemia: which, when, for whom?. Nat Rev Clin Oncol.

[7] Apperley J (2007). Part I: mechanisms of resistance to imatinib in chronic myeloid leukaemia. Lancet Oncol.

[8] Zabriskie M, Eide C, Tantravahi S, Vellore N, Estrada J, Nicolini F, Khoury H, Larson R, Konopleva M, Cortes J (2014). BCR-ABL1 compound mutations combining key kinase domain positions confer clinical resistance to ponatinib in Ph chromosome-positive leukemia. Cancer Cell.

[9] Hoemberger M, Pitsawong W, Kern D (2020). Cumulative mechanism of several major imatinib-resistant mutations in Abl kinase. Proc Natl Acad Sci USA.

[10] Lübking A, Dreimane A, Sandin F, Isaksson C, Märkevärn B, Brune M, Ljungman P, Lenhoff S, Stenke L, Höglund M (2019). Allogeneic stem cell transplantation for chronic myeloid leukemia in the TKI era: population-based data from the Swedish CML registry. Bone Marrow Transplant.

[11] Hehlmann R, Berger U, Pfirrmann M, Heimpel H, Hochhaus A, Hasford J, Kolb H, Lahaye T, Maywald O, Reiter A (2007). Drug treatment is superior to allografting as first-line therapy in chronic myeloid leukemia. Blood.

[12] Luo Z, Gao M, Huang N, Wang X, Yang Z, Yang H, Huang Z, Feng W (2019). Efficient disruption of bcr-abl gene by CRISPR RNA-guided FokI nucleases depresses the oncogenesis of chronic myeloid leukemia cells. J Exp Clin Cancer Res.

[13] Yang H, Zhou H, Huang Z, Tao K, Huang N, Peng Z, Feng W (2020). Induction of CML-specific immune response through cross-presentation triggered by CTP-mediated BCR-ABL-derived peptides. Cancer Lett.

[14] Burslem G, Schultz A, Bondeson D, Eide C, Savage Stevens S, Druker B, Crews C (2019). Targeting BCR-ABL1 in chronic myeloid leukemia by PROTAC-mediated targeted protein degradation. Can Res.

[15] Holyoake T, Helgason G (2015). Do we need more drugs for chronic myeloid leukemia?. Immunol Rev.

[16] Lu R, Hwang Y, Liu I, Lee C, Tsai H, Li H, Wu H (2020). Development of therapeutic antibodies for the treatment of diseases. J Biomed Sci.

[17] Weiner G (2015). Building better monoclonal antibody-based therapeutics. Nat Rev Cancer.

[18] Clift D, McEwan W, Labzin L, Konieczny V, Mogessie B, James L, Schuh M (2017). A method for the acute and rapid degradation of endogenous proteins. Cell.

[19] Oke V, Wahren-Herlenius M (2012). The immunobiology of Ro52 (TRIM21) in autoimmunity: a critical review. J Autoimmun.

[20] Reymond A, Meroni G, Fantozzi A, Merla G, Cairo S, Luzi L, Riganelli D, Zanaria E, Messali S, Cainarca S (2001). The tripartite motif family identifies cell compartments. EMBO J.

[21] Bottermann M, James L (2018). Intracellular antiviral immunity. Adv Virus Res.

[22] Foss S, Watkinson R, Sandlie I, James L, Andersen J (2015). TRIM21: a cytosolic Fc receptor with broad antibody isotype specificity. Immunol Rev.

[23] Rhodes D, Isenberg D (2017). TRIM21 and the function of antibodies inside cells. Trends Immunol.

[24] Singh K, Ejaz W, Dutta K, Thayumanavan S (2019). Antibody delivery for intracellular targets: emergent therapeutic potential. Bioconjug Chem.

[25] Slastnikova T, Ulasov A, Rosenkranz A, Sobolev A (2018). Targeted intracellular delivery of antibodies: the state of the art. Front Pharmacol.

[26] Qin X, Yu C, Wei J, Li L, Zhang C, Wu Q, Liu J, Yao S, Huang W (2019). Rational design of nanocarriers for intracellular protein delivery. Adv Mater (Deerfield Beach Fla).

[27] Sousa F, Castro P, Fonte P, Kennedy P, Neves-Petersen M, Sarmento B (2017). Nanoparticles for the delivery of therapeutic antibodies: Dogma or promising strategy?. Expert Opin Drug Deliv.

[28] Approval Package for Lupron Depot, 4 Months, 30 mg, Leuprolide Acetate https://www.accessdata.fda.gov/drugsatfda_docs/nda/97/020517_s002ap.pdf.

[29] Wang Y, Qin B, Xia G, Choi S (2021). FDA's poly(lactic-co-glycolic acid) research program and regulatory outcomes. AAPS J.

[30] Zhu Z, Li Y, Yang X, Pan W, Pan H (2017). The reversion of anti-cancer drug antagonism of tamoxifen and docetaxel by the hyaluronic acid-decorated polymeric nanoparticles. Pharmacol Res.

[31] Wu J, Zhou X, Li P, Lin X, Wang J, Hu Z, Zhang P, Chen D, Cai H, Niessner R (2021). Ultrasensitive and simultaneous SERS detection of multiplex microRNA using fractal gold nanotags for early diagnosis and prognosis of hepatocellular carcinoma. Anal Chem.

[32] Li M, Sun X, Zhang N, Wang W, Yang Y, Jia H, Liu W (2018). NIR-activated polydopamine-coated carrier-free "nanobomb" for in situ on-demand drug release. Adv Sci (Weinheim, Baden-Wurttemberg, Germany).

[33] Liu Q, Chen X, Jia J, Zhang W, Yang T, Wang L, Ma G (2015). pH-Responsive poly(d, l-lactic-co-glycolic acid) nanoparticles with rapid antigen release behavior promote immune response. ACS Nano.

[34] Cui Y, Xu Q, Davoodi P, Wang D, Wang C (2017). Enhanced intracellular delivery and controlled drug release of magnetic PLGA nanoparticles modified with transferrin. Acta Pharmacol Sin.

[35] Dilnawaz F, Singh A, Sahoo S (2012). Transferrin-conjugated curcumin-loaded superparamagnetic iron oxide nanoparticles induce augmented cellular uptake and apoptosis in K562 cells. Acta Biomater.

[36] Tortorella S, Karagiannis T (2014). Transferrin receptor-mediated endocytosis: a useful target for cancer therapy. J Membr Biol.

[37] Chang J, Jallouli Y, Kroubi M, Yuan X, Feng W, Kang C, Pu P, Betbeder D (2009). Characterization of endocytosis of transferrin-coated PLGA nanoparticles by the blood-brain barrier. Int J Pharm.

[38] Zhu HL, Liu T, Meng WT, Jia YQ (2007). Establishment of an imatinib resistance cell line K562R and its resistant principia. Sichuan Da Xue Xue Bao Yi Xue Ban.

[39] Heuser J, Anderson R (1989). Hypertonic media inhibit receptor-mediated endocytosis by blocking clathrin-coated pit formation. J Cell Biol.

[40] Iqbal M, Zafar N, Fessi H, Elaissari A (2015). Double emulsion solvent evaporation techniques used for drug encapsulation. Int J Pharm.

[41] Yu M, Zheng J (2015). Clearance pathways and tumor targeting of imaging nanoparticles. ACS Nano.

[42] Wei G, Rafiyath S, Liu D (2010). First-line treatment for chronic myeloid leukemia: dasatinib, nilotinib, or imatinib. J Hematol Oncol.

[43] Wang H, Tsourkas A (2019). Cytosolic delivery of inhibitory antibodies with cationic lipids. Proc Natl Acad Sci USA.

[44] Guo K, Li J, Tang J, Tan C, Hong C, Al-Aidaroos A, Varghese L, Huang C, Zeng Q (2011). Targeting intracellular oncoproteins with antibody therapy or vaccination. Sci Transl Med.

[45] Markman J, Rekechenetskiy A, Holler E, Ljubimova J (2013). Nanomedicine therapeutic approaches to overcome cancer drug resistance. Adv Drug Deliv Rev.

[46] Zhang J, Xiong H, Cao J, Wang S, Guo X, Lin B, Zhang Y, Zhao J, Wang Y, Zhang T (2018). A cell-penetrating whole molecule antibody targeting intracellular HBx suppresses hepatitis B virus via TRIM21-dependent pathway. Theranostics.

[47] Yamazaki T, Biswas M, Kosugi K, Nagashima M, Inui M, Tomono S, Takagi H, Ichimonji I, Nagaoka F, Ainai A (2020). A novel gene delivery vector of agonistic anti-radioprotective 105 expressed on cell membranes shows adjuvant effect for DNA immunization against influenza. Front Immunol.

[48] Mendonça L, Moreira J, de Lima M, Simões S (2010). Co-encapsulation of anti-BCR-ABL siRNA and imatinib mesylate in transferrin receptor-targeted sterically stabilized liposomes for chronic myeloid leukemia treatment. Biotechnol Bioeng.

[49] Mello J, Moraes V, Watashi C, da Silva D, Cavalcanti L, Franco M, Yokaichiya F, de Araujo D, Rodrigues T (2016). Enhancement of chlorpromazine antitumor activity by Pluronics F127/L81 nanostructured system against human multidrug resistant leukemia. Pharmacol Res.

[50] Fan L, Liu C, Hu A, Liang J, Li F, Xiong Y, Mu C (2020). Dual oligopeptides modification mediates arsenic trioxide containing nanoparticles to eliminate primitive chronic myeloid leukemia cells inside bone marrow niches. Int J Pharm.

[51] Järås M, Johnels P, Hansen N, Agerstam H, Tsapogas P, Rissler M, Lassen C, Olofsson T, Bjerrum O, Richter J (2010). Isolation and killing of candidate chronic myeloid leukemia stem cells by antibody targeting of IL-1 receptor accessory protein. Proc Natl Acad Sci USA.

[52] Warfvinge R, Geironson L, Sommarin M, Lang S, Karlsson C, Roschupkina T, Stenke L, Stentoft J, Olsson-Strömberg U, Hjorth-Hansen H (2017). Single-cell molecular analysis defines therapy response and immunophenotype of stem cell subpopulations in CML. Blood.

