# Supplementary figures and images for: Intracellular delivery of anti-BCR/ABL antibody by PLGA nanoparticles suppresses the oncogenesis of chronic myeloid leukemia cells
Source: J Hematol Oncol. 2021 Sep 6;14:139. doi: 10.1186/s13045-021-01150-x (PMC8422775; doi:10.1186/s13045-021-01150-x)

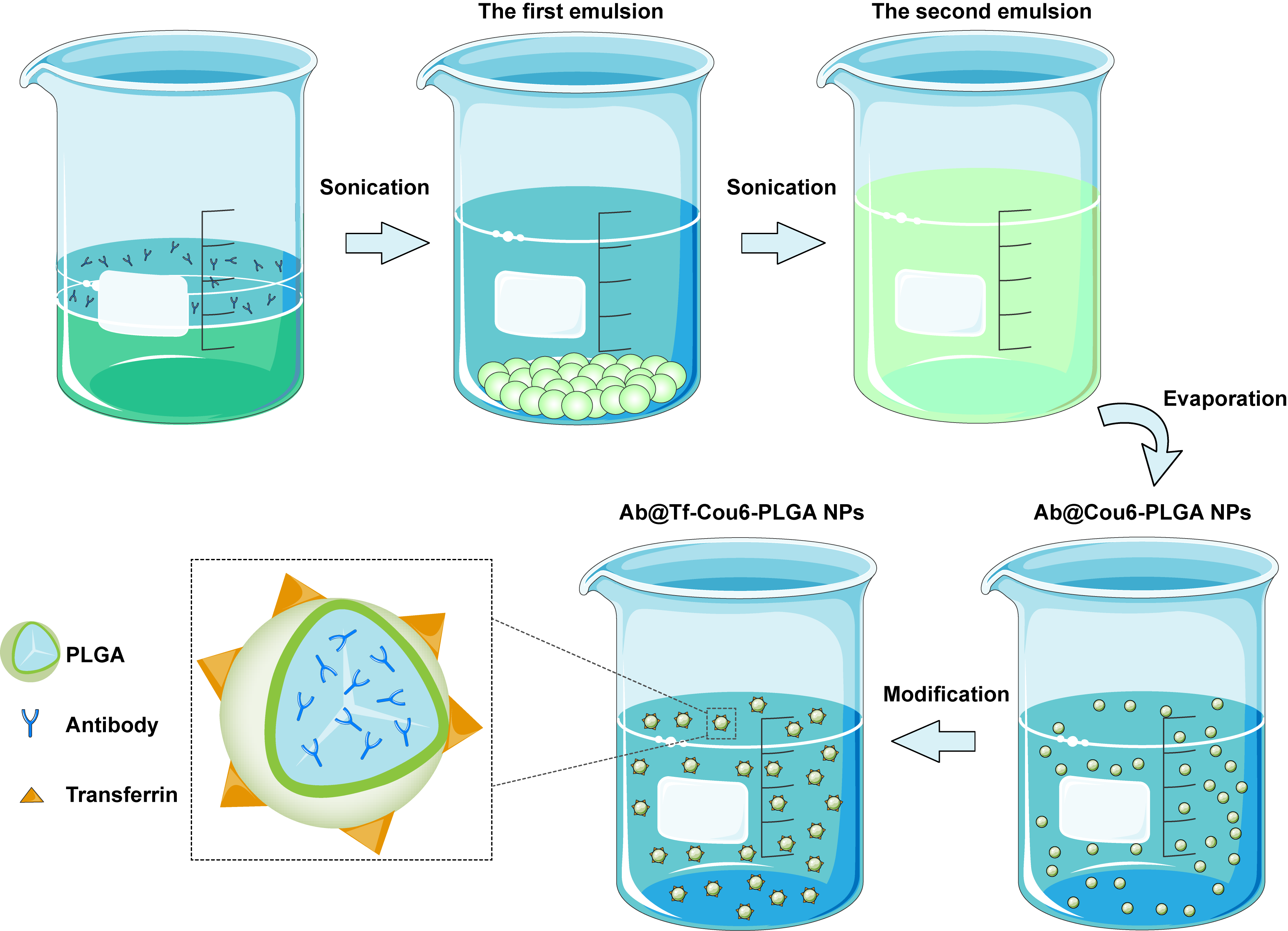

Supplement: Supplementary file 1 — Additional file 1: Fig. S1. Schematic of the double emulsion solvent evaporation method to formulate Ab@Tf-Cou6-PLGA NPs. The anti-BCR/ABL antibodies were mixed with chloroform containing PLGA and Cou6. The mixture was sonicated to obtain the first emulsion. Then, the first emulsion was mixed with 10 ml of PVA (1%) and sonicated to obtain the second emulsion. Furthermore, the chloroform was evaporated at room temperature to obtain the Ab@Cou6-PLGA NPs. The Ab@Tf-Cou6-PLGA NPs were synthesized after being modified with transferrin. [file 13045_2021_1150_MOESM1_ESM.tif]

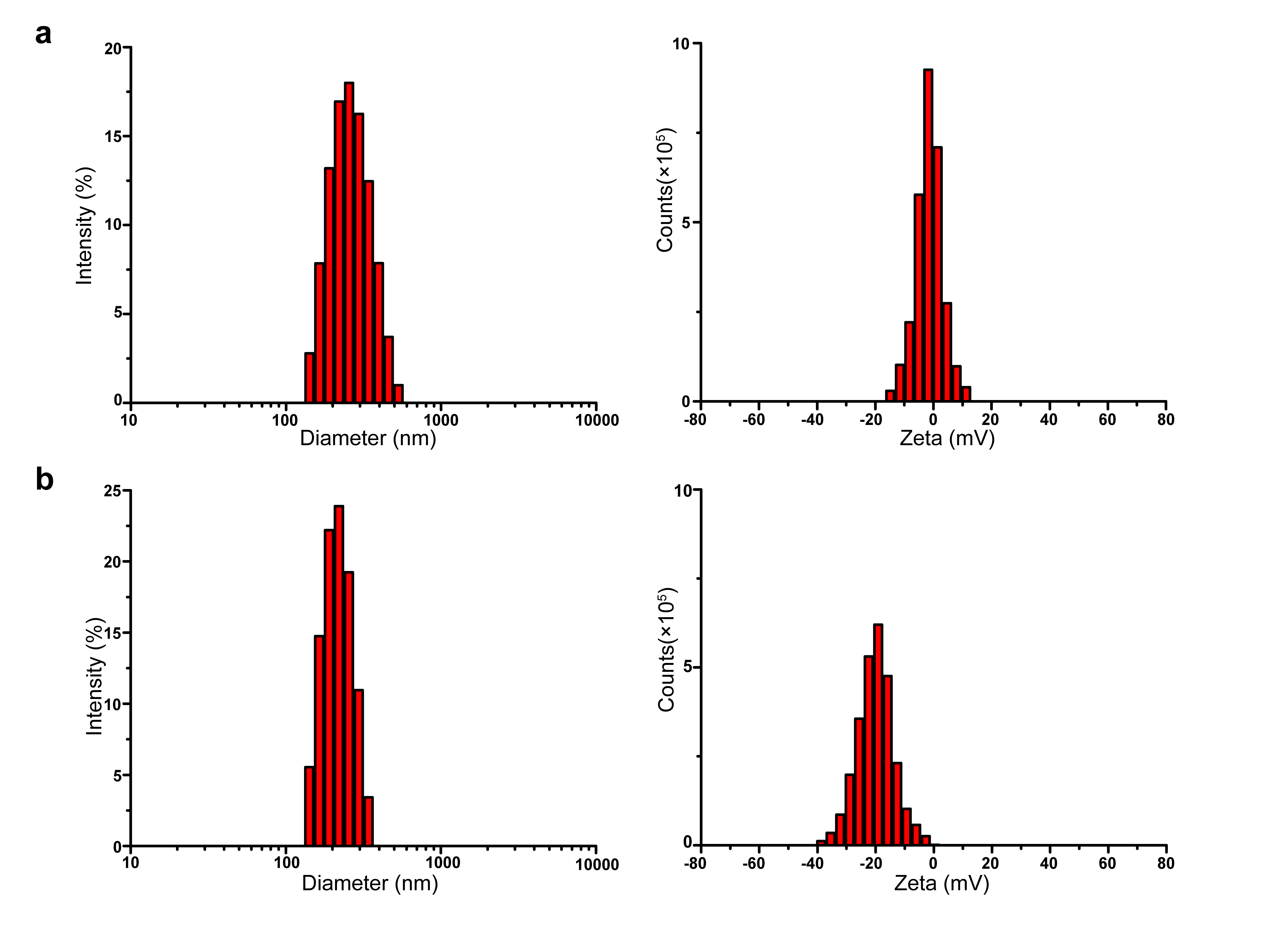

Supplement: Supplementary file 2 — Additional file 2: Fig. S2. Synthesis and characteristics of nanoparticles. (a) Diameter and Zeta potential of Ab@Cou6-PLGA NPs. (b) Diameter and Zeta potential of Ab@Tf-PLGA NPs. [file 13045_2021_1150_MOESM2_ESM.tif]

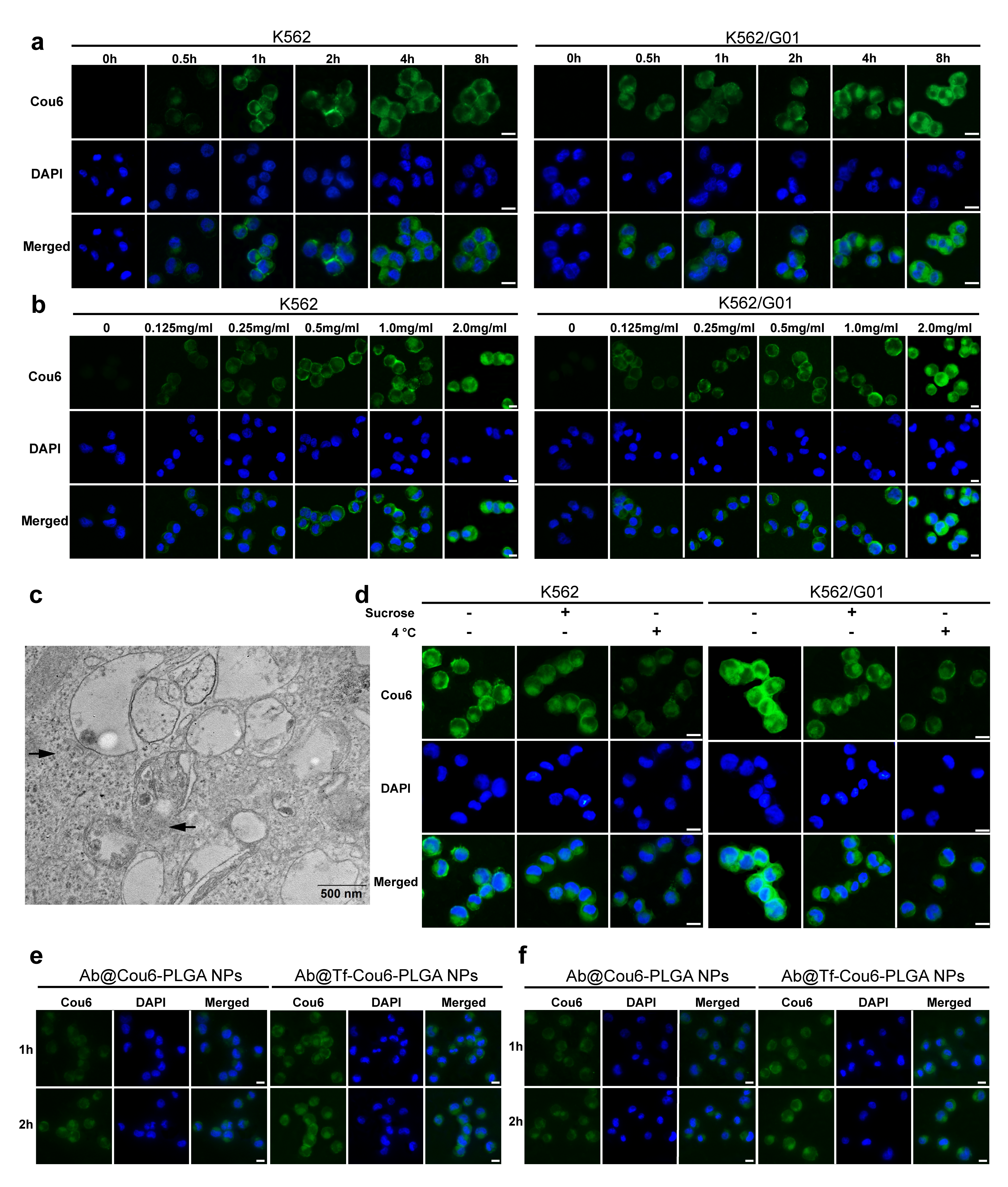

Supplement: Supplementary file 3 — Additional file 3: Fig. S3. The cellular uptake of nanoparticles. (a) Fluorescence images of Ab@Tf-Cou6-PLGA NPs uptake into CML cells in a time-dependent manner. Scale bar, 10 μm. (b) Fluorescence images of intercellular uptake of Ab@Tf-Cou6-PLGA NPs in K562 and K562/G01 cells at various doses. Scale bar, 10 μm. (c) TEM images of cellular uptake of nanoparticles. The blank arrows indicate nanoparticles. Scale bar, 500 nm. (d) Fluorescence images of cellular internalization of Ab@Tf-Cou6-PLGA NPs in K562 and K562/G01 cells after Sucrose (0.45 mM) and 4 °C treatment. Scale bar, 10 μm. (e) Fluorescence images of cellular uptake of Tf-targeted and non-targeted nanoparticles in K562 cells. Scale bar, 10 μm. (f) Fluorescence images of cellular uptake of Tf-targeted and non-targeted nanoparticles in K562/G01 cells. Scale bar, 10 μm. [file 13045_2021_1150_MOESM3_ESM.tif]

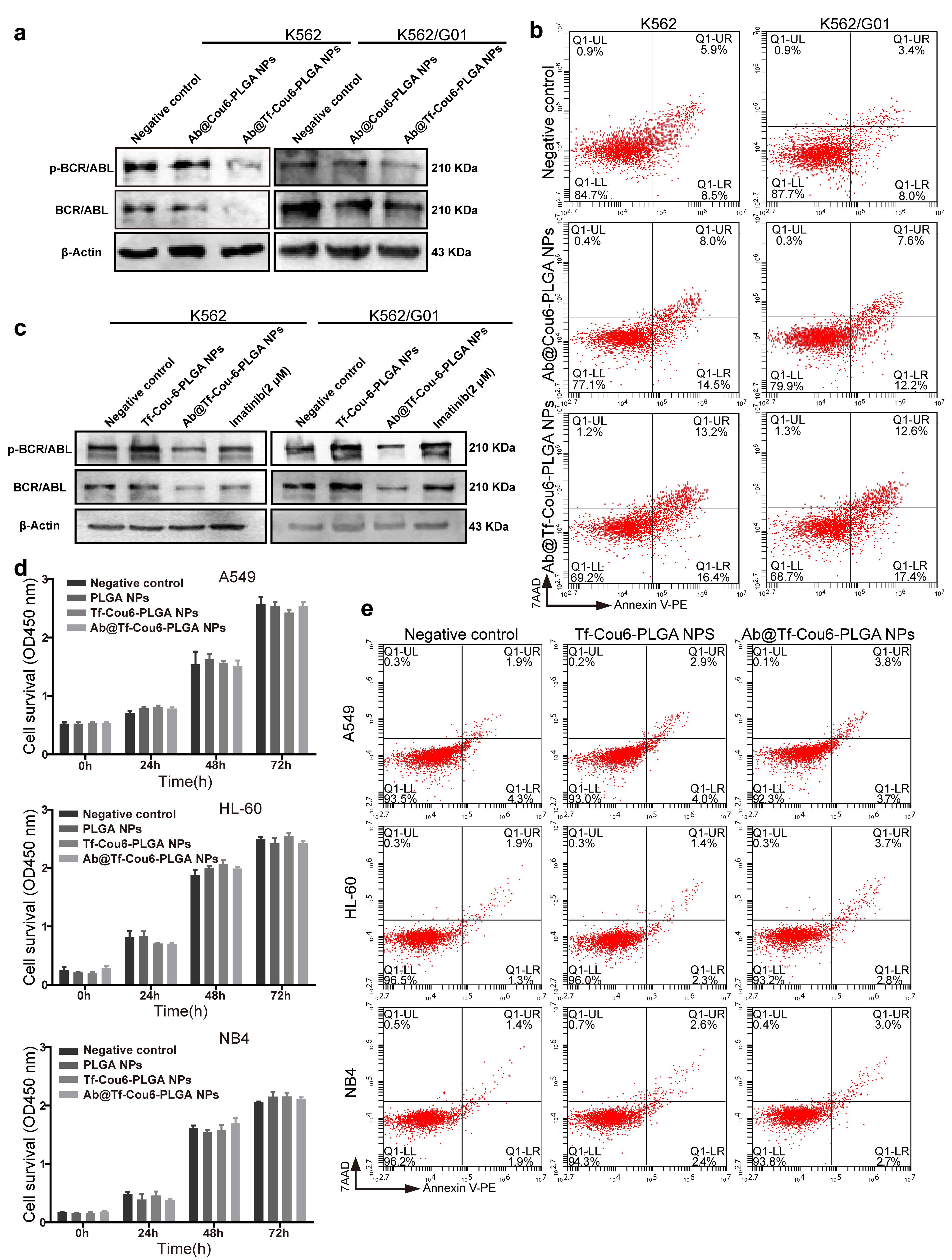

Supplement: Supplementary file 4 — Additional file 4: Fig. S4. Expression of BCR/ABL oncoprotein in nanoparticles treated CML cells. (a) The BCR/ABL and p-BCR/ABL expression level in Tf-targeted or non-targeted nanoparticles treated CML cells. (b) The apoptosis rate of CML cells after treated for 48h by Ab@Cou6-PLGA NPs or Ab@Tf-Cou6-PLGA NPs was detected by FCM. (c) The BCR/ABL expression level in CML cells after being treated by Ab@Tf-Cou6-PLGA NPs or imatinib. (d) The effect of nanoparticles on BCR/ABL negative cells was detected by CCK-8. (e) The apoptosis rate of BCR/ABL negative cells after being treated for 48h by nanoparticles was detected by FCM. [file 13045_2021_1150_MOESM4_ESM.tif]

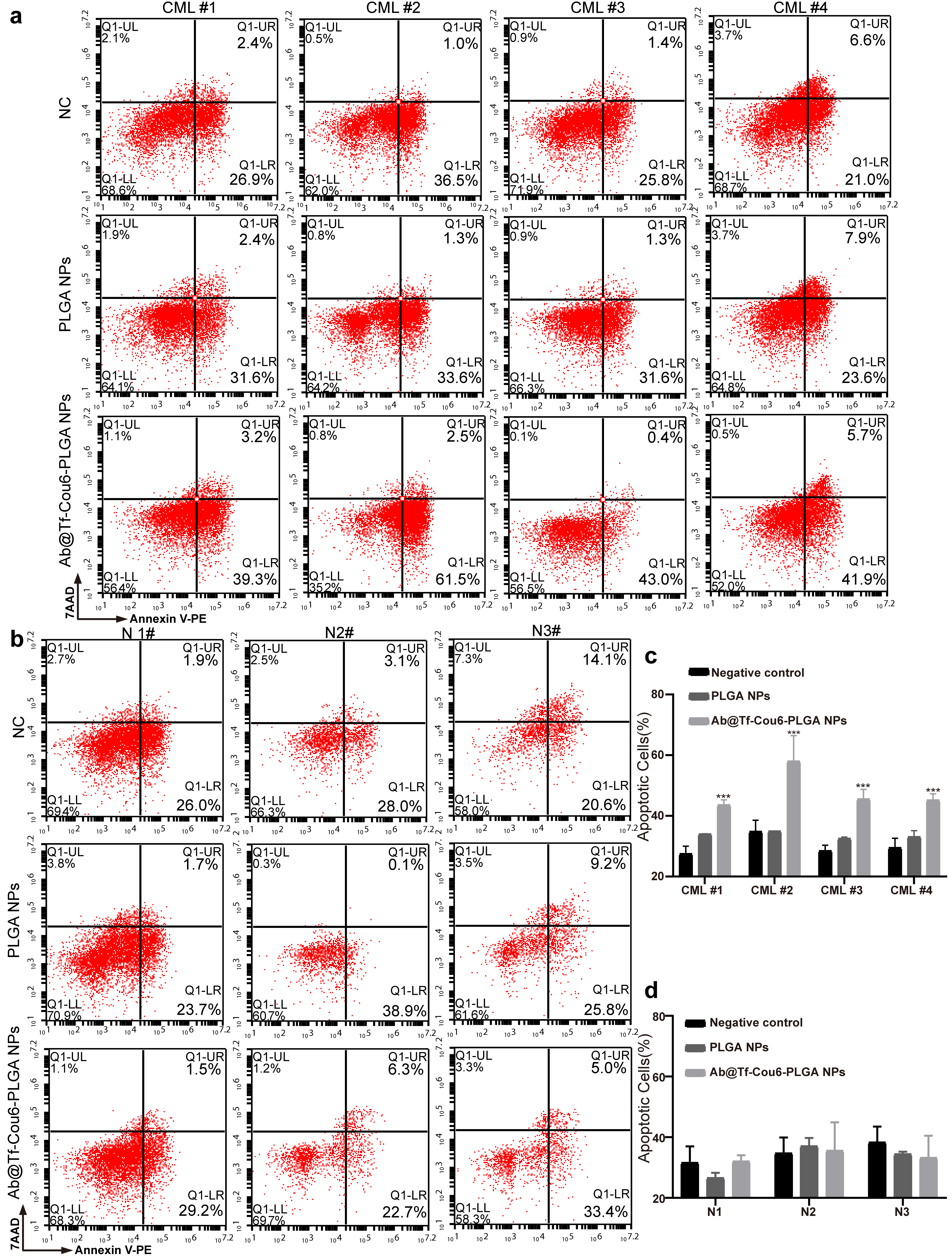

Supplement: Supplementary file 5 — Additional file 5: Fig. S5. The apoptosis was induced by Ab@Tf-Cou6-PLGA NPs in cells from CML patients. (a, c) The apoptosis rate of cells from CML patients was tested by FCM. (b, d) The apoptosis rate of cells from BCR/ABL negative donors was tested by FCM. Data are presented as the means ± SD. *P < 0.05, **P < 0.01, ***p < 0.001, ****p < 0.0001. [file 13045_2021_1150_MOESM5_ESM.tif]

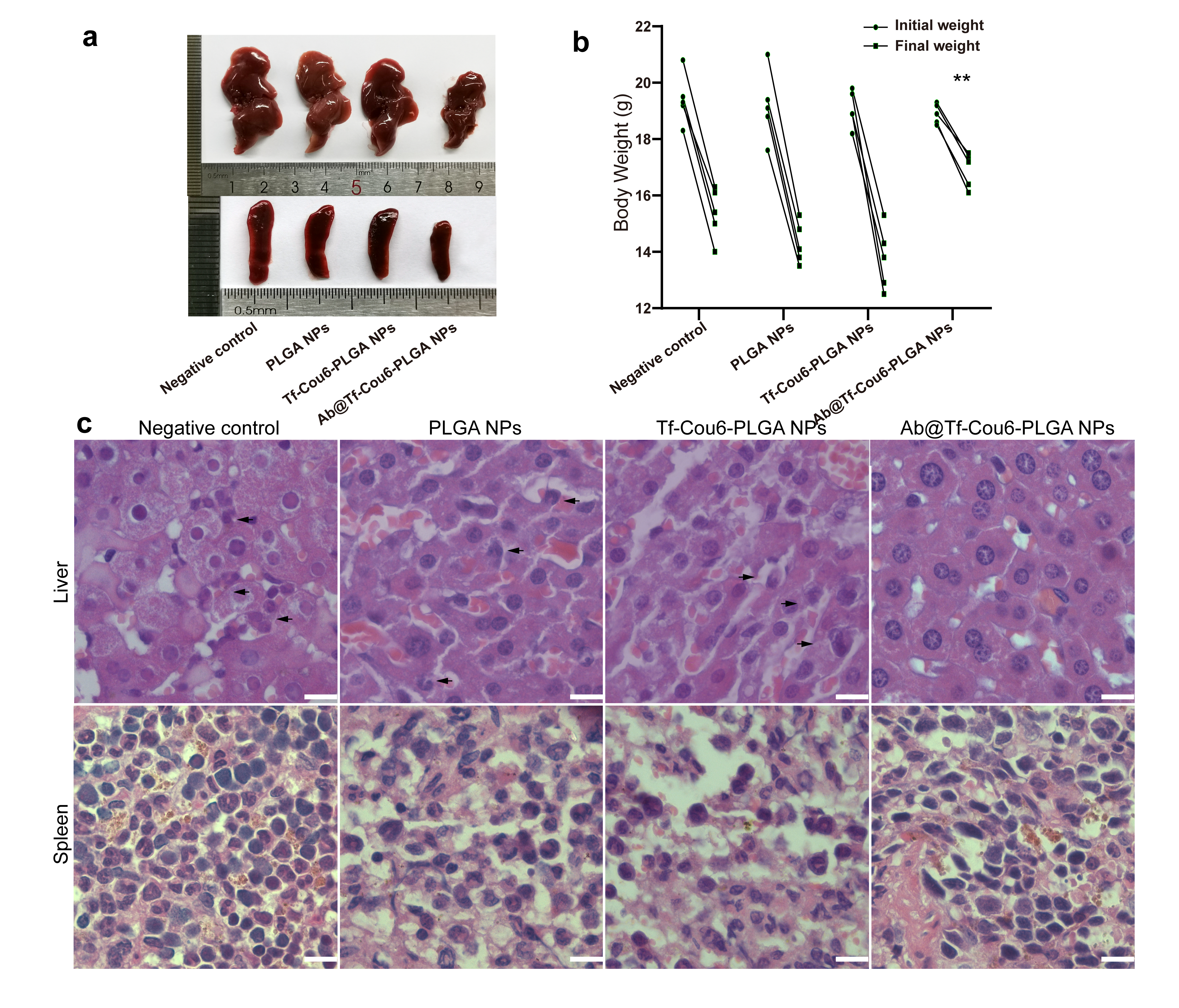

Supplement: Supplementary file 6 — Additional file 6: Fig. S6. The oncogenesis of CML cells in vivo was impaired by Ab@Tf-Cou6-PLGA NPs. (a) Images of livers and spleens form each group. (b) The initial weight and final weight of mice were recorded of each mouse. (c) The infiltration leukemic cells in the spleens and livers were analyzed by HE staining. The black arrows indicate leukemic cells. The black arrows indicate leukemic cells. Scale bar, 10 μm. Data are presented as the means ± SD. *P < 0.05, **P < 0.01, ***p < 0.001, ****p < 0.0001. [file 13045_2021_1150_MOESM6_ESM.tif]

**Supplement tables**


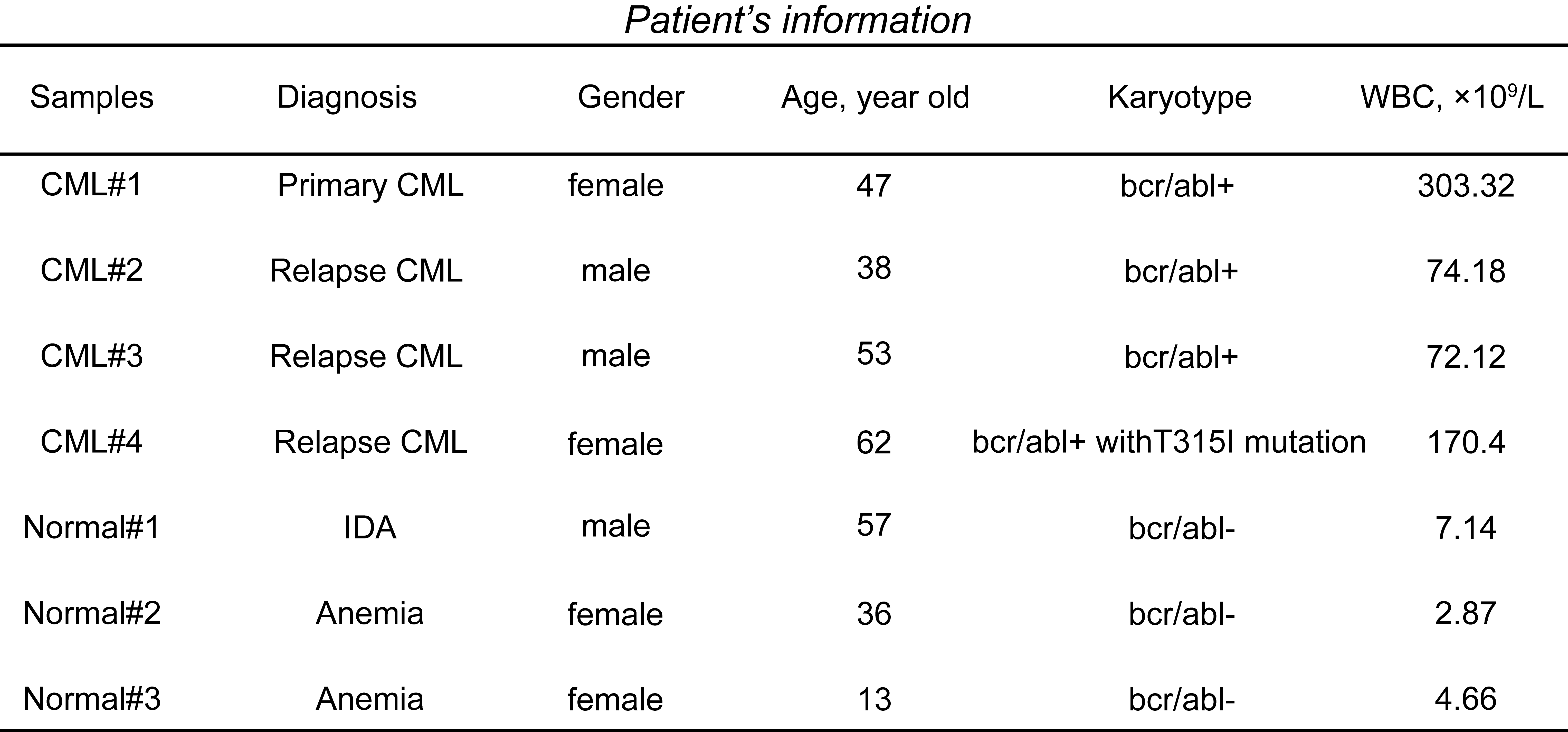


Supplement table 1


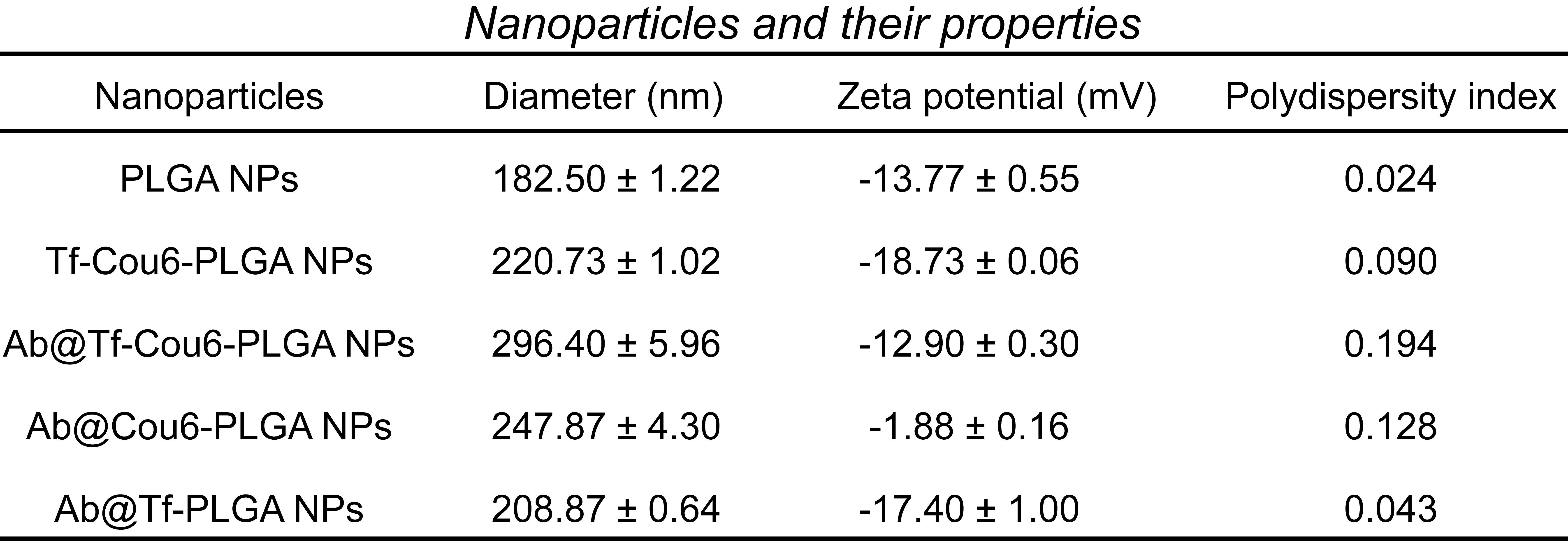


Supplement table 2


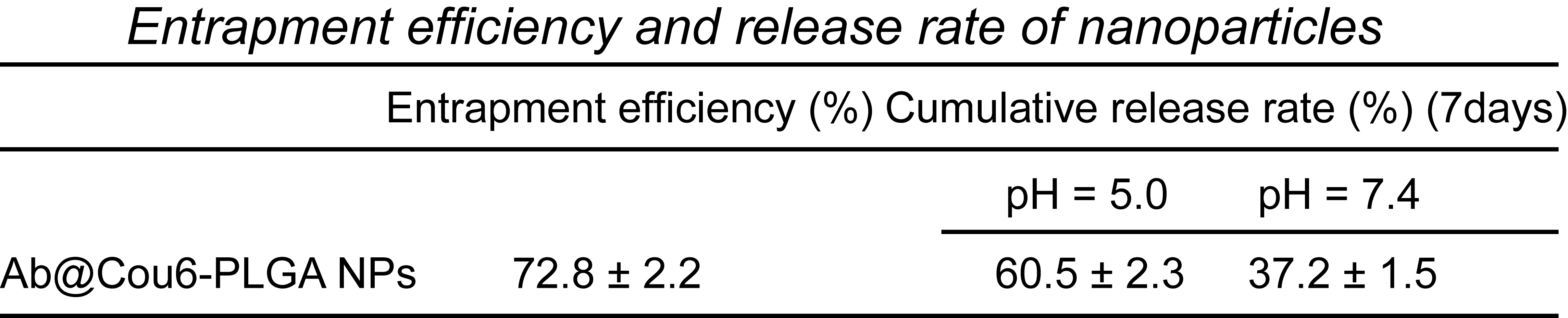


Supplement table 3

Supplement: Supplementary file 7 — Additional file 7: Supplement tables.Table S1. Patient’s information. Table S2. Nanoparticles and their properties. Table S3. Entrapment efficiency and release rate of nanoparticles. [file 13045_2021_1150_MOESM7_ESM.docx]
